# 12th International Conference on Conservative Management of Spinal Deformities – SOSORT 2015 Annual Meeting: Katowice, Poland, 7-9 May 2015

**DOI:** 10.1186/s13013-016-0077-8

**Published:** 2016-08-23

**Authors:** Eric Parent, Alan Richter, Angelo Gabriele Aulisa, Vincenzo Guzzanti, Paolo Pizzetti, Andrea Poscia, Lorenzo Aulisa, Ane Simony, Steen Bach Christensen, Mikkel O. Andersen, Alessandra Negrini, Sabrina Donzelli, Laura Maserati, Fabio Zaina, Jorge H Villafane, Stefano Negrini, Carole Fortin, Erin Grunstein, Hubert Labelle, Stefan Parent, Debbie Ehrmann Feldman, Edmond Lou, Rui Zheng, Doug Hill, Andreas Donauer, Melissa Tilburn, Jim Raso, Sanja Schreiber, Eric Parent, Greg Kawchuk, Douglas Hedden, Judith Sánchez-Raya, Antonia Matamalas Adrover, Elisabetta D’Agata, Joan Bagó Granell, Marek Kluszczynski, Anna Kluszczyńska, Jacek Wąsik, Marta Motow-Czyż, Adam Kluszczyński, Ane Simony, Karen Hojmark Hansen, Hanne Thomsen, Mikkel Meyer Andersen, Morten Vuust, Irmina Blicharska, Jacek Durmała, Bartosz Wnuk, Małgorzata Matyja, Andrzej Szopa, Małgorzata Domagalska-Szopa, Weronika Gallert-Kopyto, Tomasz Łosień, Ryszard Plintla, Franz Landauer, Karl Vanas, Gozde Gur, Necdet Sukru Altun, Yavuz Yakut, Piotr Gawda, Piotr Majcher, Lior Neuhaus Sulam, Michael Bradley, David Glynn, Alex Hughes, Erika Maude, Christine Pilcher, Andrea Lebel, Victoria Ashley Lebel, Judit Orbán, Agnieszka Stępień, Krzysztof Graff, D. Speers, Angelo Gabriele Aulisa, Vincenzo Guzzanti, Giuseppe Mastantuoni, Andrea Poscia, Lorenzo Aulisa, Angelo Gabriele Aulisa, Vincenzo Guzzanti, Francesco Falciglia, Andrea Poscia, Lorenzo Aulisa, Nikos Karavidas, Mohammadreza Etemadifar, Sabrina Donzelli, Fabio Zaina, Monia Lusini, Salvatore Minnella, Luca Balzarini, Stefano Respizzi, Stefano Negrini, Kathrin Güttinger, Jacek Durmała, Irmina Blicharska, Agnieszka Drosdzol–Cop, Violetta Skrzypulec–Plinta, Elisabetta D’Agata, Judith Sánchez-Raya, Judith Sánchez-Raya, Elisabetta D’Agata, Sławomir Paśko, Wojciech Glinkowski, Jakub Michoński, Katarzyna Walesiak, Anna Pakuła, Robert Sitnik, Wojciech Glinkowski, Helmut Diers, Piotr Majcher, Piotr Gawda, Andrea Lebel, Victoria Ashley Lebel, Piet van Loon, Ruud van Erve, Andre Grotenhuis, Karina Zapata, Eric Parent, Dan Sucato, Krzysztof Korbel, Mateusz Kozinoga, Łukasz Stoliński, Tomasz Kotwicki, Andrea Lebel, Victoria Ashley Lebel, Helmut Diers, Hagit Berdishevsky, Hagit Berdishevsky

**Affiliations:** 1 Department of Physical Therapy, University of Alberta, Edmonton, Alberta Canada; 2 U.O.C. of Orthopedics and Traumatology, Children’s Hospital Bambino Gesù, Institute of Scientific Research, P.zza S. Onofrio 4, Rome, Italy; 3 U.O.C. of Orthopedics and Traumatology, Children’s Hospital Bambino Gesù, Institute of Scientific Research, P.zza S. Onofrio, Rome 4, Italy; 4 University of Cassino, Cassino, Italy; 5 Independent practitioner, Rome, Italy; 6 Institute of public health, University Hospital “Agostino Gemelli”, Catholic University of the Sacred Heart School of Medicine, Rome, Italy; 7 Department of Orthopedics, University Hospital “Agostino Gemelli”, Catholic University of the Sacred Heart School of Medicine, Rome, Italy; 8 Sector for Spine Surgery & Research, Middelfart Hospital, Middelfart, Denmark; 9 ISICO Italian Scientific Spine Institute, Milan, Italy; 10 University of Brescia, Brescia, Italy; 11 IRCCS Don Gnocchi, Milan, Italy; 12 Université de Montréal, Research centre CHU Sainte-Justine, Montréal, Canada; 13 Université de Montréal, Institut de Recherche en santé publique de l’Université de Montréal, Montréal, Canada; 14 University of Alberta, Alberta, Canada; 15 Alberta Health Services, Alberta, Canada; 16 University of Alberta, Edmonton, Canada; 17 University of Alberta, Alberta Health Services, Edmonton, Canada; 18 Vall d’Hebron Hospital, Barcelona, Spain; 19 “Troniny” Children Rehabilitation Center in Częstochowa, Częstochowa, Poland; 20 Institute of Physiotherapy, Jan Długosz University, Czestochowa, Poland; 21 Sector for Spine Surgery & Research, Middelfart Hospital, Middelfart, Denmark; 22 Department of Radiology, Frederikshavn Sygehus, Frederikshavn, Denmark; 23 Department of Mathematics and Statistics, Aalborg University, Aalborg, Denmark; 24 School of Health Sciences in Katowice, Medical University of Silesia, Chair and Department of Rehabilitation, Katowice, Poland; 25 Chair of Kinesitherapy and Special Methods of Physiotherapy, Academy of Physical Education, Katowice, Poland; 26 School of Health Sciences in Katowice, Medical University of Silesia in Katowice, Department of Physiotherapy, Katowice, Poland; 27 School of Health Sciences in Katowice, Medical University of Silesia in Katowice, Department of Medical Rehabilitation, Katowice, Poland; 28 School of Health Sciences in Katowice, Medical University of Silesia in Katowice, Department of Physiotherapy, Katowice, Poland; 29 School of Health Sciences in Katowice, Medical University of Silesia in Katowice, Department of Adapted Physical Activity and Sport, Katowice, Poland; 30 University Clinic of Orthopedics (PMU), Salzburg, Austria; 31 Hacettepe University, Çankaya, Ankara, Turkey; 32 Akay Hospital, Ankara, Turkey; 33 Uniwersytet Medyczny w Lublinie, Lublin, Poland; 34 The Israeli Scoliosis Center, Tel Aviv, Israel; 35 Scoliosis SOS Clinic, London, Great Britain; 36 University of York, York, Great Britain; 37 Ottawa & District Physiotherapy Clinic, Scoliosis Physiotherapy and Posture Centre, McLeod Street, Ottawa, K2P 0Z8 Canada; 38 Saba University School of Medicine, Saba, Dutch Caribbean Netherlands; 39 Józef Piłsudski University of Physical Education, Warsaw, Poland; 40 Scheck and Siress, Chicago, IL USA; 41 U.O.C. of Orthopedics and Traumatology, Children’s Hospital Bambino Gesù, Institute of Scientific Research, P.zza S. Onofrio 4, Rome, Italy; 42 Institute of public health, University Hospital “Agostino Gemelli”, Catholic University of the Sacred Heart School of Medicine, Rome, Italy; 43 Department of Orthopedics, University Hospital “Agostino Gemelli”, Catholic University of the Sacred Heart School of Medicine, Rome, Italy; 44 U.O.C. of Orthopedics and Traumatology, Children’s Hospital Bambino Gesù, Institute of Scientific Research, P.zza S. Onofrio 4, Rome, Italy; 45 U.O.C. of Orthopedics and Traumatology, Children’s Hospital Bambino Gesù, Institute of Scientific Research, P.zza S. Onofrio 4, Rome, Italy; 46 University of Cassino, Cassino, Italy; 47 Institute of public health, University Hospital “Agostino Gemelli”, Catholic University of the Sacred Heart School of Medicine, Rome, Italy; 48 Department of Orthopedics, University Hospital “Agostino Gemelli”, Catholic University of the Sacred Heart School of Medicine, Rome, Italy; 49 Scoliosis Spine Laser Centre, Athens, Greece; 50 Orthopedic Department, Esfahan University of Medical Sciences, Isfahan, Iran; 51 ISICO Italian Scientific Spine Institute, Milan, Italy; 52 ICH Istituto Clinico Humanitas, Milan, Italy; 53 IRCCS Don Gnocchi, Milan, Italy; 54 University of Brescia, Brescia, Italy; 55 Zürcher Hochschule für angewandte Wissenschaften (ZHAW), Winterthur, Switzerland; 56 School of Health Sciences in Katowice, Medical University of Silesia, Chair and Department of Rehabilitation, Katowice, Poland; 57 School of Health Sciences in Katowice, Medical University of Silesia, Chair of Woman’s Health, Katowice, Poland; 58 Vall d’Hebron Hospital Institute, Barcelona, Spain; 59 Vall d’Hebron Hospital, Barcelona, Spain; 60 Vall d’Hebron Hospital, Barcelona, Spain; 61 Research Institut Vall d’Hebron Hospital, Barcelona, Spain; 62 Warsaw University of Technology, Institute of Micromechanics and Photonics, Warsaw, Poland; 63 Chair and Department of Orthopedics and Traumatology of the Locomotor System, Baby Jesus Clinical Hospital, Center of Excellence “TeleOrto” for Telediagnostics and Treatment of Disorders and Injuries of the Locomotor System, Medical University of Warsaw, Warsaw, Poland; 64 Warsaw University of Technology, Warsaw, Poland; 65 Department of Orthopaedics and Traumology of Locomotor System, Center of Excellence “TeleOrto”, Medical University of Warsaw, Warsaw, Poland; 66 Research & Development, Schlangenbad, Germany; 67 Uniwersytet Medyczny w Lublinie, Lublin, Poland; 68 Ottawa & District Physiotherapy Clinic, Scoliosis Physiotherapy and Posture Centre, McLeod Street, Ottawa, K2P 0Z8 Canada; 69 Saba University School of Medicine, Saba, Dutch Caribbean Netherlands; 70 Care to Move Orthopedics, Deventer, The Netherlands; 71 Neurosurgery, UMC Radboud University, Nijmegen, The Netherlands; 72 Texas Scottish Rite Hospital, Dallas, Texas USA; 73 University of Alberta, Edmonton, Canada; 74 Rehasport Clinic, Poznań, Poland; 75 Spine Disorders Unit-Departament of Pediatric Orthopaedics and Traumatology, Poznań University of Medical Sciences, Poznań, Poland; 76 Ottawa & District Physiotherapy Clinic, Scoliosis Physiotherapy and Posture Centre, McLeod Street, Ottawa, K2P 0Z8 Canada; 77 Saba University School of Medicine, Saba, Dutch Caribbean Netherlands; 78 Research & Development, Schlangenbad, Germany; 79 SchrothNYC, New York, NY USA; 80 SchrothNYC, New York, NY USA

## Abstract

O1 The functional properties of paraspinal muscles in adolescents with idiopathic scoliosis (AIS): A systematic review of the literature

Eric Parent, Alan Richter

O2 The importance of the lateral profile in the treatment of idiopathic scoliosis

Angelo Gabriele Aulisa, Vincenzo Guzzanti, Paolo Pizzetti, Andrea Poscia, Lorenzo Aulisa

O3 Radiological outcome in Adolescent idiopathic scoliosis patients 20 years after treatment

Ane Simony, Steen Bach Christensen, Mikkel O Andersen

O4 Junctional Kyphosis, how can we detect and monitor it during growth?

Alessandra Negrini, Sabrina Donzelli, Laura Maserati, Fabio Zaina, Jorge H Villafane, Stefano Negrini

O5 Usefulness of the clinical measure of trunk imbalance in adolescent idiopathic scoliosis

Carole Fortin, Erin Grunstein, Hubert Labelle, Stefan Parent, Debbie Ehrmann Feldman

O6 Can ultrasound imaging be used to determine curve flexibility when designing spinal orthoses?

Edmond Lou, Rui Zheng, Doug Hill, Andreas Donauer, Melissa Tilburn, Jim Raso

O7 Reliability of the Schroth curve type classification in adolescents with idiopathic scoliosis (AIS)

Sanja Schreiber, Eric Parent, Greg Kawchuk, Douglas Hedden

O8 Can Trunk Appearance Perception Scale (TAPS) be used as a descriptive tool of scoliosis severity?

Judith Sánchez-Raya, Antonia Matamalas Adrover, Elisabetta D’Agata, Joan Bagó Granell

O9 Magnitude of the Cobb angle on an X-ray in relation to the angle of trunk rotation in children who come to the “Troniny” Scoliosis Treatment Centre

Marek Kluszczynski, Anna Kluszczyńska, Jacek Wąsik, Marta Motow-Czyż, Adam Kluszczyński

O10 Cobb angel measurement without X-ray, a novel method

Ane Simony, Karen Hojmark Hansen; Hanne Thomsen; Mikkel Meyer Andersen; Morten Vuust

O11 The postural tone magnitude and distribution in patients diagnosed with an adolescent idiopathic scoliosis: a preliminary study

Irmina Blicharska, Jacek Durmała, Bartosz Wnuk, Małgorzata Matyja

O12 From studies on the function of the respiratory system in children with body posture defects

Andrzej Szopa, Małgorzata Domagalska-Szopa, Weronika Gallert-Kopyto, Tomasz Łosień, Ryszard Plintla

O13 Scoliosis as the “first” sign of various diseases

Franz Landauer, Karl Vanas

O14 The effectiveness of core stabilization exercises versus conventional exercises in addition to brace wearing in patients with adolescent idiopathic acoliosis

Gozde Gur, Necdet Sukru Altun, Yavuz Yakut

O15 The effect of physiotherapy techniques on the body balance in patients with scoliosis treated with corrective appliances

Piotr Gawda, Piotr Majcher

O16 New combine method treating AIS – preliminary results

Lior Neuhaus Sulam

O17 Does a 4-week intensive course of ScolioGold therapy reduce angle of trunk rotation in scoliotic patients: a retrospective case series.

Michael Bradley, David Glynn, Alex Hughes, Erika Maude, Christine Pilcher

O18 Schroth physiotherapy method without bracing is an effective treatment for scoliosis in improving curves and avoiding surgery and should be offered as a treatment option for scoliosis in Canada: case series

Andrea Lebel, Victoria Ashley Lebel, Judit Orbán

O19 Rotation of the trunk and pelvis and coupled movements in the sagittal plane in double support stance in adolescent girls with idiopathic scoliosis

Agnieszka Stępień, Krzysztof Graff

O20 Curve progression analysis in Risser 0 patients orthotically managed with compliance monitors

D. Speers

O21 Conservative treatment in Scheuermann’s kyphosis: comparison between lateral curve and variation of the vertebral geometry

Angelo Gabriele Aulisa, Vincenzo Guzzanti, Giuseppe Mastantuoni, Andrea Poscia, Lorenzo Aulisa

O22 The plaster cast in the conservative treatment of idiopathic scoliosis can still play a positive role?

Angelo Gabriele Aulisa, Vincenzo Guzzanti, Francesco Falciglia, Andrea Poscia, Lorenzo Aulisa

O23 Bracing for Adolescent Idiopathic Scoliosis (AIS) and Scheuermann Kyphosis : The issue of overtreatment in Greece

Nikos Karavidas

O24 Efficacy of Milwaukee brace for correction of scheurmann kyphosis

Mohammadreza Etemadifar

O25 The three dimensional analysis of the Sforzesco brace correction

Sabrina Donzelli, Fabio Zaina, Monia Lusini, Salvatore Minnella, Luca Balzarini, Stefano Respizzi, Stefano Negrini

O26 Quality of Life in adolescents with idiopathic scoliosis: A comparison measured by the Kidscreen 27 between scoliotic patients and healthy controls

Kathrin Güttinger

O27 The degree of illness acceptance in young women with idiopathic scoliosis treated with orthopedic braces: a preliminary study

Jacek Durmała, Irmina Blicharska, Agnieszka Drosdzol–Cop, Violetta Skrzypulec–Plinta

O28 Which are the personality traits of the patients with Adolescent Idiopathic Scoliosis?

Elisabetta D’Agata, Judith Sánchez-Raya

O29 How many Scolioses do exist in the same person? A zoom vision on the perception of the patient

Judith Sánchez-Raya, Elisabetta D’Agata

P1 The algorithm for the automatic detection of the pelvic obliquity based on analysis of the PA viev of the x-ray image

Sławomir Paśko, Wojciech Glinkowski

P2 Monitoring of spine curvatures and posture during pregnancy using surface topography – case study and method assessment

Jakub Michoński, Katarzyna Walesiak, Anna Pakuła, Robert Sitnik, Wojciech Glinkowski

P3 Spinal rotation under static and dynamic conditions: a prospective study comparing normative data vs. scoliosis

Helmut Diers

P4 The principle of non-surgical treatment of idiopathic scoliosis right-sided breast depending on the volatility of the formation of the intervertebral discs and vertebral bodies

Piotr Majcher, Piotr Gawda

P5 Unexpected late progression of adolescent idiopathic scoliosis treated with short-term, aggressive, full-time bracing and Schroth physiotherapy with excellent preliminary result: case study

Andrea Lebel, Victoria Ashley Lebel

P6 Visible posture in relation to the neuroanatomical and neurodynamical features in spinal deformations

Piet van Loon, Ruud van Erve, Andre Grotenhuis

P7 Immediate effects of scoliosis-specific corrective exercises on the Cobb angle after 1 week and after 1 year of practice

Karina Zapata, Eric Parent, Dan Sucato

P8 Retrospective analysis of idiopathic scoliosis medical records coming from one out-patient clinic for compatibility with Scoliosis Research Society criteria of brace treatment studies

Krzysztof Korbel, Mateusz Kozinoga, Łukasz Stoliński, Tomasz Kotwicki

P9 Adult female with severe progressive scoliosis possibly secondary to benign tumor removal at age 3 treated with scoliosis specific Schroth physiotherapy after refusing surgery: case study

Andrea Lebel, Victoria Ashley Lebel

P10 New aspects of scoliosis therapy planning and monitoring

Helmut Diers

P11 Outcome of intensive outpatient rehabilitation in an adult patient with M. Scheuermann evaluated by radiologic imaging – a case report

Hagit Berdishevsky

P12 The effectiveness of a Scoliosis Specific Home Exercise Program and bracing to reduce an idiopathic scoliosis curve with more than 90 % success in less than a year of exercises. Case report.

Hagit Berdishevsky

## ORAL PRESENTATIONS

### O1 The functional properties of paraspinal muscles in adolescents with idiopathic scoliosis (AIS): A systematic review of the literature

#### Eric Parent, Alan Richter

##### Department of Physical Therapy, University of Alberta, Edmonton, Alberta, Canada

**Introduction:** Exercise-based approaches exist; however, it is unclear whether these approaches are based on scientific findings in the literature on trunk muscle deficits in scoliosis that could be targeted by exercises. The aims of this study were to systematically review the literature to understand the functional muscular properties of paraspinal muscles in AIS to determine: 1) differences in functional outcomes between patients with AIS and controls, 2) differences in functional outcomes between sides (concave and convex) in patients compared to controls 3) differences between concave and convex sides as well as levels in subjects with AIS, 4) differences in functional outcomes between different curve types. 5) Associations between functional outcomes and curve characteristics, and 6) associations between functional characteristics and progression.

**Design:** Systematic review

**Methods:** A search was conducted in EMBASE, MEDLINE, SPORTdiscus, CINAHL, SCOPUS, and Web of Science, for keywords describing functional properties of paraspinal muscles and measurement tools including: scoliosis, spinal deformity, spinal muscles, erector, rotatores, longissimus, spinalis, illiocostalis, force, strength, endurance, fatigability, and muscle fatigue. Two reviewers independently reviewed abstracts and then full-text articles to determine if they met selection criteria. Two reviewers used an extraction form to extract information and appraise the quality during the full-text review. Levels of evidence were determined for summarized results for each of the 6 objectives.

**Results:** Our search yielded 316 unique records. Inter-reviewer agreement for abstract selection was Kappa = 0.73 and was 0.77 for full-text inclusion. Full-text review was done for 48 papers and 24 were included. A large amount of heterogeneity was observed in sample studied and assessment methodology. Quality appraisal revealed that no study met a minimum of 50 % of the relevant quality criteria. Studies recruited consistently low sample sizes and samples were largely heterogeneous. Limited evidence was noted supporting, a prolonged bilateral EMG activation during gait between AIS and controls; elevated heterolateral:homolateral activity ratios during side-bending; overall weakness in those with scoliosis compared to controls; no asymmetry in normalized muscle activity during submaximal isometric contractions; prolonged latencies on the side of the spine opposite of the curve and bilaterally in response to an unloading reflex; strength & muscle volume differences are most commonly pronounced in double curves; Axial rotation of the UEV is correlated with a high convex:concave activity ratio at the LEV; no correlation between latency and curve severity, but a correlation between latency and progression and higher convex:concave EMG ratios and progression, this is more pronounced in sitting positions. 

**Conclusions:** Evidence is limited on most of our six objectives due to low quality evidence and lack of research about muscle impairments in scoliosis. Current exercise-based interventions cannot yet be said to be based on a strong understanding of muscle impairments in scoliosis. Research is needed using large, homogenous samples allowing for a comparison between curve types and examining relation to the risk of progression. While many exercise-based programs focus on addressing endurance deficits using high repetitions and long holds, no studies were found on endurance deficits in AIS.

### O2 The importance of the lateral profile in the treatment of idiopathic scoliosis

#### Angelo Gabriele Aulisa^1^, Vincenzo Guzzanti^2^, Paolo Pizzetti^3^, Andrea Poscia^4^, Lorenzo Aulisa^5^

##### ^1^U.O.C. of Orthopedics and Traumatology, Children's Hospital Bambino Gesù, Institute of Scientific Research, P.zza S. Onofrio 4, Rome, Italy; ^2^U.O.C. of Orthopedics and Traumatology, Children's Hospital Bambino Gesù, Institute of Scientific Research, P.zza S. Onofrio, Rome 4, Italy ; University of Cassino, Cassino, Italy; ^3^Independent practitioner, Rome, Italy; ^4^Institute of public health, University Hospital "Agostino Gemelli", Catholic University of the Sacred Heart School of Medicine, Rome, Italy; ^5^Department of Orthopedics, University Hospital "Agostino Gemelli", Catholic University of the Sacred Heart School of Medicine, Rome, Italy

**Background**

Adolescent idiopathic scoliosis (AIS) is a 3-dimensional spinal deformity. Thoracic sagittal malalignment has been thought to play an important role in the development of thoracic idiopathic scoliosis.

Thoracic hypokyphosis with increasing axial rotational instability is claimed to be a primary factor for the initiation of Idiopathic Scoliosis (IS) according to some authors. Other authors have shown that Thoracic hypokyphosis is strongly associated with curve progression in thoracic IS.

Moreover, the studies evaluating the impact of hypokyphosis on conservative treatment are limited in number and fragmentary.

**Design**

In previous studies, we have evaluated the impact of rotation on the conservative treatment of idiopathic scoliosis.

The purpose of the present study was to determine the trend of hypokyphosis during conservative treatment and its interaction with the lateral curve and rotation.

**Material and methods**

From a prospective database, we selected all patients with adolescent thoracic idiopathic scoliosis, with Risser 0-3 and lateral radiographs performed at the beginning and at the end of treatment.From this group, we excluded all cases in which X-rays did not allow a correct measurement of kyphosis angle.

107 patient with Lyon brace, 97 female and 10 male, mean age 12.4 ± 1.81 years fulfilled the inclusion criteria.

The minimum follow-up was 24 months.

Postero-Anterior and lateral radiographs were used to estimate the lateral curve magnitude (CM), the torsion of the apical vertebra (TA) and the degree of kyphosis (KM) at 2 time points: beginning of treatment (t1) and 2-year minimum follow-up from the end of treatment (t5). Three outcomes were distinguished in agreement with SRS criteria: correction, stabilization and progression.

Statistical analyses was performed.

**Results**

The results from our study showed that of the 107 patients CM mean value was 34.5 ± 9.9 SD at t1 and 21.7 ± 12.5 SD at t5.

The difference in cobb degrees between t5-t1 was -12.8° (*p* < 0.01) in CM, -4.5° (*p* < 0.01) in TA and -2.0°(*p* < 0.01) in KM.

The regression analysis shows that the evolution of the scoliosis is not associated with the initial level of kyphosis, both in terms of Cobb (*p* = 0.31; R2 = 0.009) and Pedriolle degrees (*p* = 0.74; R2 = 0.001). Confirms, furthermore, that the evolution of the scoliosis is not associated with the evolution of kyphosis, both in terms of Cobb (*p* = 0.989; R2 = 0.000)and Pedriolle degrees(*p* = 0.788; R2 = 0.001).

Curve correction was accomplished in 87 patients (81.3 %), whereas a curve stabilization was obtained in 17 patients (15.8 %). 3 patients (3 %) had a curve progression and of these, in only one surgical treatment was recommended.

**Discussion**

Our results confirm that a well-designed brace with correctly push can limit the feared worsening of hypo-kyphosis.

Moreover, contrary to what is commonly thought, it doesn’t appear that the hypo-kyphosis modifies the results of conservative treatment.

It is confirmed, instead, as reported in previous papers both clinical and biomechanical: rotation significantly affects the outcome of conservative treatment.

A rotation of more than 20 °, leads to the hysteresis of the intervertebral discs, hinders the transmission of the corrective forces to the vertebrae and leads to any brace corrective action.

### O3 Radiological outcome in Adolescent idiopathic scoliosis patients 20 years after treatment

#### Ane Simony, Steen Bach Christensen, Mikkel O Andersen

##### Sector for Spine Surgery & Research, Middelfart Hospital, Middelfart, Denmark

**Purpose**

The purpose of this study was to evaluate the long term radiological outcome, curve progression and adjacent level degeneration 20 years after scoliosis treatment.

**Method**

219 patients treated with Boston brace or posterior spinal fusion a. m Harrington were invited to participate in a long term evaluation with clinical examination and x-ray evaluation. The old medical charts and x-ray descriptions where available.Standing X-ray was examined, the Cobb angel measured and compared to the patient’s prior x-rays and the adjacent levels where evaluated for any signs of adjacent level disease or local kyphosis.

**Results**

159 patients participated (78 %). 66 patients treated with Boston brace and 92 patients, treated with posterior spinal fusion a.m. Harrington from 1983-1990 at University Hospital Copenhagen.In the Brace group, the Cobb angel prior to treatment was 37.5° (35.1°-40.0°), after treatment 34.7° (31.9°-37.5°). Cobb angel after 20 years was 40.2° (36.7°-43.6°).In the surgical group the Cobb angel prior to treatment was 54.5° (50.4°-58.8°), 1 year postoperative 29.5 ° (25.7°-33.9°).Cobb angel after 20 years 32.35° (27.9°-39.5°).26 patients had distal segment degeneration in x-rays (16.5 %), 4 patients treated with Brace and 22 patients with posterior spinal fusion.8 patients had proximal segment degeneration (5 %), 2 treated with brace and 6 patients with posterior spinal fusion. 4 patients were treated with posterior fusion of the distal adjacent segment (2.6 %), 1 treated with Brace and 3 treated with posterior spinal fusion.

**Conclusion**

The average follow up was 24.5 years (range 24-31 years). The Brace group had a small reduction of the spinal deformity during the treatment period, and X-rays shows a small progression of the deformity, Cobb angel increasing 5.5° within 20 years. The surgically treated patients had a large correction during surgery and there is no progression or loss of correction over a 20 year period.Only 4 patients in the Brace group have distal segment degeneration and 1 was treated with a one level spinal fusion.The surgically treated groups had a significant deformity correction during surgery and have maintained the correction after 20 years. 22 patients have distal degeneration and 3 patients were treated with distal adding on surgery.

### O4 Junctional Kyphosis, how can we detect and monitor it during growth?

#### Alessandra Negrini^1^, Sabrina Donzelli^1^, Laura Maserati^2^, Fabio Zaina^1^, Jorge H Villafane^3^, Stefano Negrini^2,3^

##### ^1^ISICO Italian Scientific Spine Institute, Milan, Italy; ^2^University of Brescia, Brescia, Italy; ^3^IRCCS Don Gnocchi, Milan, Italy

**Introduction**

Despite its importance in affecting adult pain, and disability, there is a lack of universal criteria for the diagnosis and evaluation of junctional kyphosis (JK) and a gold standard measurement and diagnostic system does not exist.

**Aim**

To verify the sensibility and specificity of clinical, and Formetric data in identifying junctional kyphosis in respect to the radiographical standard references.

**Material and methods**

***Design****:* This is a cross sectional study from a prospective database started in March 2003.

*Participants:* 52 patients: 29 with JK, and 23 with thoracic hyperkyphosis (TK).

*Inclusion criteria*: patients affected by JK or TK at first visit with a complete clinical, radiographical and surface topography evaluation.

*Groups.* JK: lower limit of kyphosis below T12. Control group: subjects with a thoracic kyphosis radiographic measure exceeding 50°Cobb.

*Diagnostic tests used to detect JK:*

- Clinical: plumbline distances: T12 < S1.
- Formetric criteria included the % of thoraco-lumbar inflexion point in trunk length over 60 %.

*Statistics:* sensitivity, specificity, positive (PPV) and negative predictive values (NPV), by using diagnostic test vs the actual gold standard were calculated using a 2x2 table.

**Results**

The sensititvity of the plumbline distances of T12 < S1, in detecting JK in respect to radiographic criteria, resulted 55 %, with an accuracy of 46 %. The specificity of the test was 65 %, PPV 67 % and NPV 33 %.

The sensitivity of the surface topography test resulted 73 %, as of the 29 patients with a JK x-rays diagnosis 22 showed a positive test, and only 7 without JK resulted negative. Therefore the specificity of the test was only 32 %. PPV and NPV resulted respectively of 40 % and 59 %.

**Conclusion**

The need for a useful criteria able to characterize JK to allow diagnosis and monitoring of the deformity is still lacking, and further studies will deepen this issue.

### O5 Usefulness of the clinical measure of trunk imbalance in adolescent idiopathic scoliosis

#### Carole Fortin^1^, Erin Grunstein^1^, Hubert Labelle^1^, Stefan Parent^1^, Debbie Ehrmann Feldman^2^

##### ^1^Université de Montréal, Research centre CHU Sainte-Justine, Montréal, Canada; ^2^Université de Montréal, Institut de Recherche en santé publique de l’Université de Montréal, Montréal, Canada

**Introduction**

Trunk imbalance, defined as a shift of the trunk in the frontal plane and measured with a plumbline from C7 to S1, is part of the clinical evaluation in adolescent idiopathic scoliosis (AIS). To date, little is known about evidences of this clinical measure in AIS.

**Objectives**

1) To determine the reliability and validity of the clinical measure of trunk imbalance, 2) to assess its prevalence and 3) to explore the relationship between trunk imbalance and Cobb angle and with back pain in adolescents with AIS.

**Materials and methods**

Trunk imbalance measurements of 55 participants aged 10 to 19 years old with AIS (Cobb angle: 15° to 60°) were assessed by a physical therapist on two separate occasions. Markers placed on spinous processes of C7 and S1 were used to measure the horizontal distance between the plumbline placed at C7 and S1 with a rigid ruler. Cobb angle and trunk imbalance were measured on radiographs taken on the same day and the pain level was determined using the Numerical Pain Rating Scale (NPRS) and the Scoliosis Research Society-22 (SRS-22) pain score. Generalizability theory (f) was used to estimate the reliability and standard error of measurement (SEM) for the overall, test**-**retest design. Prevalence of trunk imbalance was given in percentage using the cut-off of 6.1 mm (minimal detectable change value). Pearson correlation coefficients (r) were used to assess validity of trunk imbalance compared with the radiographic method and to explore the association with Cobb angle and back pain. Logistic regression models also served to describe trunk imbalance (as a dichotomous outcome using several cutoffs: 10 mm, 15 mm and 20 mm) as a function of back pain.

**Results**

Trunk imbalance measured with a plumbline demonstrated high test**-**retest reliability (f : 0 .98 and SEM : 2.2 mm) and good correlation with measurements on radiographs (r :0.83, *p* : < 0.005).Trunk imbalance prevalence was 85 %. We found fair to moderate significant positive correlation between trunk imbalance and Cobb angle (r : 0.32 to 0.66, *p* < 0.05) but not with back pain. In the logistic regression model, there was a trend for trunk imbalance > 20 mm to be related with lower back pain.

**Conclusions**

The good psychometric properties and the high prevalence of trunk imbalance provide evidence for the usefulness of this clinical measure in AIS. Trunk imbalance can be easily measured in a clinical setting. A longitudinal study with a larger cohort is still needed to document the association of trunk imbalance with curve progression and back pain as well as the implications of the treatment of trunk imbalance on both Cobb angle and back pain.

### O6 Can ultrasound imaging be used to determine curve flexibility when designing spinal orthoses?

#### Edmond Lou^1^, Rui Zheng^1^, Doug Hill^2^, Andreas Donauer^2^, Melissa Tilburn^2^, Jim Raso^1^

##### ^1^University of Alberta, Alberta, Canada; ^2^Alberta Health Services, Alberta, Canada

**Background**

Spinal flexibility of a patient with Adolescent Idiopathic Scoliosis (AIS) affects in-orthosis correction. More flexible spines are better corrected in an orthosis which in turn should lead to better longterm outcomes. Curently, it is difficult to know how flexible a curve is during the orthosis design stage because radiographs are not done in order to minimize radiation exposure in growing children. Recently, ultrasound spinal imaging has been shown capable of measuring proxy Cobb angles and vertebral rotations reliably and repeatably in AIS patients.

**Design and level of evidence**

This pilot study investigated ultrasound imaging as a tool to provide spine flexibility in real time to assist the orthotists goal for the final in-orthosis correction. This is a Level III of Evidence study.

**Materials and methods**

AIS subjects prescribed full-time TLSO were asked to participate to this study. The inclusion criteria used the SOSORT brace management guidelines. Local ethics approval was received and all participants signed consent forms prior to participation. During the casting clinic, participants were scanned with ultrasound in the prone position. During scanning, participants with single right thoracic curves bent maximally to the right side while keeping their hips level and both shoulders in contact with the bed. An ultrasound (US) system with built-in position tracking was used to scan the spine along the spinal processes. An in-house program was used to reconstruct, display and measure proxy Cobb angles in real-time. Spinal bending correction (flexibility) was calculated as [Pre-orthosis X-ray Cobb – US Bending Proxy Cobb] / Pre-orthosis X-ray Cobb x 100 %. The final in-orthosis correction was calculated as [Pre-orthosis Cobb - In-orthosis Cobb] / Pre-orthosis Cobb x 100 %.

**Results**

Six participants (age: 13.9 ± 1.2 years) with 9 curves were recuited. The largest treated Cobb angles measured 39° ± 7° on a posterioanterior radiograph prior to casting. Curve flexibility averaged 74 ± 12 % (range: 56 % – 82 %). During casting, the orthotist used individual flexibility measures as targets for acceptable in-orthosis correction. Approximately 8 minutes were added to the clinic visit time: 1 minute to scan, 3 minutes to process and 4 minutes to measure the parameters. Recuritment rate was 100 %. At the following in-orthosis clinic, the orthotist used acheivment of 50 % – 70 % of the curves’ flexibility to determine the need for readjustment. To date, 3 participants have returned to clinic, the major treated flexibility versus the in-orthosis correction was 77 % vs 46 %, 81 % vs 36 % and 82 % vs 57 %. None of these subjects required adjustments to the orthosis.

**Discussion & conclusion**

Ultrasound imaging has the potential to provide radiation-free real-time measures of spinal flexibility but more study is requird to validate the process before it can be widely used.

### O7 Reliability of the Schroth curve type classification in adolescents with idiopathic scoliosis (AIS)

#### Sanja Schreiber^1^, Eric Parent^1^, Greg Kawchuk^1^, Douglas Hedden^2^

##### ^1^University of Alberta, Edmonton, Canada; ^2^University of Alberta, Alberta Health Services, Edmonton, Canada

**Introduction**

Schroth exercises are scoliosis-specific exercises aiming to improve postural alignment, control and stability of the spine. A classification system with four curve types is used to guide Schroth therapists in prescribing specific exercises for patients with scoliosis. This classification should be reliable to assure appropriate therapy delivery. We developed a rule-based algorithm to assist in reliably classifying patients. The aim of this study was to determine the intra- and inter-therapist reliability in classifying patients with AIS using our proposed classification algorithm.

**Design**

An international intra- and inter-rater reliability study.

**Material and methods**

We recruited 44 consecutive volunteers with AIS, aged 10 to 18, with curves between 10°-50° from a scoliosis clinic and 10 consecutive English-speaking volunteers from the international registry of certified Schroth therapists. The patients’ standing posture from each side and the Adam’s forward bend test were videotaped by the primary author. Therapists reviewed a manual with operational definitions, rated and reviewed four practice cases streamed from the study website illustrating each of the four Schroth curve types as training for using the algorithm before the study started. After the training period, the therapists, blinded to participants’ identity, rated video assessments presented randomly on two occasions at least seven days apart. The intra- and inter-rater reliability estimates were calculated for the entire sample of therapists (*N* = 10), the therapists who reported having full understanding of the algorithm (well-trained, *N* = 6), and the therapists who had conceptualized and used the algorithm in a randomized controlled trial (experienced, *N* = 2). Gwet’s AC1 and weighted AC1 coefficients were used to calculate the reliability. A weighted analysis was justified. The 3c and 4c Schroth curve patterns share thoracic curves and balanced pelvis and their exercise prescription does not differ as drastically (assigned weight =0.5) as between 3cp vs. 4cp (opposite pelvis corrections), 4cp vs. 3c (emphasis on pelvis and lumbar vs. thoracic curves) or 3cp vs. 4c curve patterns (emphasis on pelvis and thoracic vs. thoracic and lumbar curves only), for which pairs the assigned weight was 0.

**Results**

Patient’s age was 14.2 ± 2.0 years and their mean largest Cobb angle was 25.8° ± 10.0^o^. Based on the experienced rater’s ratings, there were nine 3c, 12 3cp, six 4c and 17 4cp curve types. The overall intra-rater AC1 was 0.64 (95 % CI 0.53-0.73), 0.70 (95 % CI 0.60-0.78) among well-trained raters, and 0.81 (95 % CI 0.77-0.85) in experienced raters. The weighted intra-rater AC1 averaged 0.75 (95 % CI 0.63-0.84) overall, 0.82 (95 % CI 0.73-0.88) in well-trained raters, and 0.89 (95 % CI 0.80-0.94) in experienced raters. Inter-rater AC1 was 0.43 (95 % CI 0.28-0.58) overall, 0.50 (95 % CI 0.38-0.61) for well-trained raters, and 0.67 (95 % CI 0.50-0.85) for experienced raters. The weighted inter-rater AC1 was 0.48 (95 % CI 0.29-0.67) overall, 0.61 (95 % CI 0.49-0.72) among well-trained, and 0.79 (95 % CI 0.64-0.94) among experienced raters.

**Conclusions**

A high level of understanding of the algorithm improved the intra- and inter-rater reliability justifying future refinement of the training. Weighted analysis demonstrated adequate intra- and inter-rater reliability, suuporting usage of the proposed algorithm in raters reporting full understanding.

### O8 Can Trunk Appearance Perception Scale (TAPS) be used as a descriptive tool of scoliosis severity?

#### Judith Sánchez-Raya, Antonia Matamalas Adrover, Elisabetta D'Agata, Joan Bagó Granell

##### Vall d'Hebron Hospital, Barcelona, Spain

**Background**

The Trunk Appearance Perception Scale (TAPS) is a valid instrument for evaluating the patient perception of their trunk deformity. There are no studies that evaluate the validity of TAPS when used by doctors to assess trunk deformity.

**Design and level of evidence**

Correlational study to assess inter and intra-observer reliability for usage of TAPS instrument.

**Material and methods**

The sample consisted of 32 patients (26 females), with a mean age of 15.56 and a mean Cobb Angle of 40.17° (ranging from18° to 74°). Mean TAPS was 3.33. Patients were also given access to the TAPS instrument too.

For each patient, three photographies were made (anterior, posterior and Adam’s test position). Three specialists in scoliosis evaluated the three photographies, according to the TAPS scoring. One week later, the three evaluators assessed again the photographies.

**Results**

The mean results of the first TAPS evaluations were: 3.67 (E1), 3.67 (E2), 3.78 (E3). The ICC (average measure) result for inter-observer reliability was .89 (*p* < 0.001).

The Maximum Cobb Angle correlation was -.55 (E1), -50 (E2), -.51 (E3).

The means of the second evaluations were: 3.85 (E1), 3.71 (E2), 3.79 (E3). ICC for intra observer reliability was ICC = 0.96.

Correlation between Cobb angle and Patient TAPS was r = -.433 (p < 0.05), while no significant correlations were found between Patient TAPS and Observer TAPS evaluations. Significant and high correlations were found between Maximum Cobb Angles and Observer TAPS, ranging from r = -0.46 and r = -0.58 (*p* < 0.005).

**Conclusion**

When scored by clinicians, TAPS presented a satisfactory inter-observer and intra observer reliability and good correlation with Maximum Cobb Angle. This being said, this tool proves to be an effective and accurate tool when describing curve severity.

### O9 Magnitude of the Cobb angle on an X-ray in relation to the angle of trunk rotation in children who come to the “Troniny” Scoliosis Treatment Centre

#### Marek Kluszczynski ^1^, Anna Kluszczyńska ^1^, Jacek Wąsik ^2^, Marta Motow-Czyż ^2^, Adam Kluszczyński ^1^

##### ^1^“Troniny” Children Rehabilitation Center in Częstochowa, Częstochowa, Poland, ^2^Institute of Physiotherapy, Jan Długosz University, Częstochowa, Poland

**Introduction**

The limiting value of a back asymmetry measured in a child, authorising a therapist to send the child to have an radiograph taken, is 7^o^ ,according to the SOSORT consensus. When a child comes to a scoliosis therapy centre with a radiograph, then it is possible to determine a value of the Cobb angle and compare it to the ATR angle.

The objective of the study is evaluation of Cobb angles in a group of children with ATR angles within the 4-6^o^ range examined in the clinic for the first time. The analysis provided below is to draw attention to the rightfulness of taking up prevention and X-ray testing in the group of children with back asymmetry below 7^o^.

**Design and level of evidence**

Cross-sectional Study with verification by reference (gold) standard.

**Material and methods**

The material were 117 children (30 %) from among 351 treated in the Centre within the last 5 years, who had a radiograph of their backs taken on their medical appointment. On the basis of the radiograph of the spine, the Cobb’s angle, rotation of the vertebra, acc. to Cobb were determined and the degree of skeletal maturity was evaluated by means of the Risser sign. Moreover, the examination included: evaluation of the angle of lumbar lordosis and thoracic kyphosis with the use of the Saunders inclinometer as well as evaluation of the angle of trunk rotation (ATR) with the use of the Bunnell scoliometer. The screened children were 6-17 years of age, on average 12.6 +/- 1.9 y. o. a. . The group included 64 % girls and 36 % boys, respectively. Results of the measured Cobb angle values and ATR angle values for both groups are compared.

Materials have been statistically analysed. Contingency tables have been made and chi-square tests have been calculated. Statistically significant results was assumed to be p < 0.05.

**Results.**

The group of children with ATR angle values lower than 7^o^ included 59 % (69) members of the entire group, whereas the group of children with ATR angle values higher than 7^o^ included 41 % (48) members of the examined group. In each group, the number of children having curvatures within the 3 ranges of magnitude of the Cobb angle 10-14^o^, 15-20^o^, and over 20^o^, was determined. Thus, the group with the ATR angle lower than 7^o^ included 24 (34.8 %) children with Cobb angles 10-14^o^, 23 (33.3 %) children with Cobb angles 15-20^o^, and 22 (31.9 %) children with Cobb angles over 20^o^. The group with the ATR angle higher than 7^o^ included 10 (20.8 %) children with Cobb angles 10-14^o^, 8 (16.7 %) children with Cobb angles 15-20^o^, and 30 (62.5 %) children with Cobb angles over 20^o^. There were significant statistical differences in distribution between individual groups (Chi-square = 10.833; df = 2; *p* ° = 0.004). A higher proportion of group members within the in the 10-14^o^ and 15-20^o^ Cobb angle ranges was observed in the group of children with the ATR lower than 7^o^. There was a higher proportion of >20^o^ Cobb angles in the group of children with the ATR higher than 7^o^.

**Conclusions.**

1. The frequency of incidence of scoliosis with the value of the Cobb angle 15^o^ and higher in the group of children referred to the Scoliosis Treatment Centre due to spinal asymmetry lower than 7^o^ ATR was 37 %.
2. Decisions about starting scoliosis prophylaxis and taking a radiograph of spine of children younger than 12 years of age with diagnosed spinal asymmetry of ATR 4-6^o^ value should be considered individually.

### O10 Cobb angel measurement without X-ray, a novel method

#### Ane Simony^1^, Karen Hojmark Hansen^1^, Hanne Thomsen^2^, Mikkel Meyer Andersen^3^, Morten Vuust^2^

##### ^1^Sector for Spine Surgery & Research, Middelfart Hospital, Middelfart, Denmark; ^2^Department of Radiology, Frederikshavn Sygehus, Frederikshavn, Denmark; ^3^Department of Mathematics and Statistics, Aalborg University, Aalborg, Denmark

**Background**

X rays have been the Golden Standard for evaluation of development and progression of scoliosis, for many years. Cobb angel measurement is the most important tool, to determine curve progression and effect of treatment. The patients are children or adolescent and standard x-rays of the spine expose the breast area, the thyroid, and the gonads, with ionizing radiation. Increased incidence of cancer is observed among patients, treated for adolescent idiopathic scoliosis [1,2]. Previous studies with measurement of the trunk rotation, rotation of the spine etc. has not been to create a method to determine Cobb.

**Aim**

To validate the accuracy of The Manual Method, against convention radiographs.

**Methods**

We hypothesised, that by manually marking the spinous process and take a photograph of the full spine, we where able to measure Cobb. This study is a validation of this method, and a comparison of this method, and Cobb measurements in X-ray.130 consecutive patients, referred to standing x-ray of the spine, were invited to participate in this study. 78 patients fulfilled the inclusion criteria. Before x-ray, the Spinous processes where manually palpated from T1 to S1, and marked with a pen. The patient was placed for X-ray, and the photo was taken with the patient standing in exactly the same position, as the AP X-ray. Marking and photographs where taken by a Research nurse, and staff from Radiological Department. X-rays were evaluated by 2 independent doctors, and the photographs were evaluated by the same 2 doctors, 2 weeks later. The measurements where evaluated by an independent statistician. Inter and intra observer variation was evaluated, and the difference between X-ray and Photo was evaluated.

**Results**

For the thoracic curves, the mean difference was 6.9 (*p* value < 0.0001), such that on average, the angle measured with x-ray was 6.9 degrees larger than that measured with photo. The Pearson correlation between x-ray and photo angle was 0.58 (*p* value < 0.0001).For the thoracolumbal curves, the mean difference was 5.2 (*p* value < 0.0001). The Pearson correlation between x-ray and photo angle was 0.66 (*p* value < 0.0001).In the lumbar group, only 7 patients participated. This is not enough to evaluate the methods feasibility, and these results are not presented.

**Conclusion**

By this study, is seems possible to evaluated Cobb, by a manually method. The method has been proven successful in thoracic and the thoraco-lumbar region. Further examination is needed, to evaluate if this method is useable in the lumbar region as well.Furthermore studies with continuous measurements are needed, to ensure this method can be used to determine progression as well.

**References**

1. Bone CM, Hsieh GH. The risk of carcinogenesis from radiograhs to pediatric orthopaedic patients. J Pediatr Orthop. 2000 Mar-Apr;20(2):251-4.

2. Levy AR, Goldberg MS, Hanley JA, Mayo, Poitras B. Projecting the lifetime risk of cancer from

### O11 The postural tone magnitude and distribution in patients diagnosed with an adolescent idiopathic scoliosis: a preliminary study

#### Irmina Blicharska^1^, Jacek Durmała^1^, Bartosz Wnuk^1^, Małgorzata Matyja^2^

##### ^1^School of Health Sciences in Katowice, Medical University of Silesia, Chair and Department of Rehabilitation, Katowice, Poland; ^2^Chair of Kinesitherapy and Special Methods of Physiotherapy, Academy of Physical Education, Katowice, Poland

**Background**

The scientific researches confirm the relationship between abnormal postural patterns in infants and defects occurring in the later stages of life. During the child’s development, the central nervous system is developed and integrated. Manifestation of its functioning is reflected in postural and motor patterns as well as parameters characterizing postural tone. An accurate postural tone is strictly connected with the central nervous system functioning, the body stabilization and the relation between mobility and stability. It also determines an accurate arrangement of the body segments. Due to its disorder, there are compensation changes. By adjusting the child’s movement possibilities to his/her own abilities, conditioned by abnormally functioning CNS, it leads to the secondary, neuro-orthopedic changes in the body posture.

**Material and methods**

37 subjects of both sexes, aged x = 12.8 ± 1.53 were qualified to a prospective study. The study group (A) was constituted by patients diagnosed with an adolescent idiopathic scoliosis, who have not been treated yet. The average value of the primary curvature was x = 21.4°. The criterion for exclusion from this group was the scoliosis etiology other than unknown or intensive, regular rehabilitation. The control group (B) was formed by healthy children, who underwent a screening test that did not show any signs of scoliosis and other significant deviations within the body posture. The quantitative assessment of parameters associated with the postural tone was measured with the calculator of the magnitude and distribution of the postural tone. Twenty-one parameters, i.e. a functional length of muscle groups or the body posture components, were entered into the calculator. On the basis of such parameters, the program automatically determines the values of the Postural Tone Index (that define magnitude of postural tone), also the values of the Spastoidal Tone Index and Athetoidal Tone Index (that define distribution of the postural tone). The angle of trunk rotation (ATR) was determined with Bunell’s scoliometer. This tool was also used in a screening test (in the control group) in order to exclude the scoliosis occurrence. The body posture quality was assessed by means of Kasperczyk’s Scale.

The results were analyzed in the Statistica v.10. A compliance distribution of the variables with a normal distribution was performed with Shapiro-Wilk’s test. The correlation between parameters was determined on the basis of R-Pearson’s test. The comparison of the measurable parameters between groups was performed by means of U-Mann-Whitney’s test. The value *p* <0.05 was determined as a level of statistical significance.

**Results**

In the group of patients with scoliosis, the average value of the Postural Tone Index was x = 0.349, while in the control group x = 0.230. Values closer to 0 indicate the better quality of postural tone. The difference between groups is statistically significant (*p* <0.05). There was no correlation between the value of the Cobb’s angle in the primary curvature and the value of the Postural Tone Index.

**Conclusions**

The subjects, with the adolescent idiopathic scoliosis, demonstrated a significantly reduced postural tone. The study requires a follow-up and further supplementation. The study group is too small to assign occurring disturbances to the population of people with scoliosis.

### O12 From studies on the function of the respiratory system in children with body posture defects

#### Andrzej Szopa^1^, Małgorzata Domagalska-Szopa^2^, Weronika Gallert-Kopyto^3^, Tomasz Łosień^2^, Ryszard Plintla^4^

##### ^1^School of Health Sciences in Katowice, Medical University of Silesia in Katowice, Department of Physiotherapy, Katowice, Poland; ^2^School of Health Sciences in Katowice, Medical University of Silesia in Katowice, Department of Medical Rehabilitation, Katowice, Poland; ^3^School of Health Sciences in Katowice, Medical University of Silesia in Katowice, Department of Physiotherapy, Katowice, Poland; ^4^School of Health Sciences in Katowice, Medical University of Silesia in Katowice, Department of Adapted Physical Activity and Sport, Katowice, Poland

**Background**

Scoliosis, despite of being a deviation of spine’s anatomical axis from its mechanical axis especially in the lateral direction, also constitutes a faulty posture in the frontal plane. Although the lateral curvature dominates, multi-layered and multi-segmented nature of scoliosis is emphasized. Signs of scoliosis also pertain changes in regard to anteroposterior curvatures, rotation and torsion of vertebrae in the transversal plane, and they also directly impact the positioning and shape of other sections of the locomotor system. Numerous studies which concern with functioning of the respiratory system in scoliosis, were focused mainly on the value of the spine's lateral curvature angle.

**Design and level of evidence**

Replacing measurements of the angle value of the spine's lateral curvature with analysis of body posture based on the moiré topography (MT) allows the assessment of nearly all scoliosis symptoms combined with spirometric measurements. The purpose of presented study was to provide answers to the following questions:

1. Do restrictive ventilatory defects occur in children with scoliosis?

2. Which features of body posture have a decisive influence on the functioning of the respiratory system?

**Material and methods**

The study involved 68 children with idiopathic scoliosis, aged 9–15. All subjects met the following criteria: (1) older than 7 years of age, (2) able to follow verbal directions, (3) mild scoliosis (angle of vertebral lateral curvature < 25°), (4) no previous surgical procedures. Basic elements of the conducted research included: body posture examination, based on moiré topography (MT) in standing and spirometric measurement. The MT examination was performed by using a CQ Electronic System (Poland). The spirometric examination was performed on a Micro Lab MK8 Viasys spirometer, while chest expansion was assessed by means of chest circumference measurement using measuring tape.

**Results**

For the majority of patients with mild scoliosis, the obtained results, including the values of the basic ventilation indicator (i.e. the percentage of due lung vital capacity- VC%), were within norm and did not confirm the existence of features characteristic for restrictive ventilatory defects. Moreover, no dependency was stated between basic functioning parameters of the respiratory system, and features characteristic for scoliosis, i.e. the primary curvature angle value and degree of rotation. Meanwhile, the obtained results indicated that in these children the VC values depend on the shape of thoracic kyphosis, determined by: depth (TKD), length (TKL) as well as the length/depth indicator (TKI%). The calculated correlation coefficients (r- Pearson) showed that the ventilation efficiency was not deteriorated due to deepened thoracic kyphosis but, to the contrary, VC% decreased together with shallowing of the curvature, which usually accompanies scoliosis.

**Conclusions**

Research results, as well as their analyses presented in this study, are not meant to negate the existence of function defects of the respiratory system in idiopathic scoliosis, but merely provide some evidence which undermine the unambiguity of presented problem.

### O13 Scoliosis as the “first” sign of various diseases

#### Franz Landauer, Karl Vanas

##### University Clinic of Orthopedics (PMU), Salzburg, Austria

**Background**

The success or failure of any brace treatment is closely linked with thecause of scoliosis and concomitant diseases.

**Design and level of evidence**

This observational study is designed like a case control study to differentiate causes of scoliosis.

**Material and methods**

285 patients referred as adolescent “idiopathic” scoliosis and indicated for bracing (SOSORT criteria) were followed over 5 years. They were searched at every appointment for changes in the spine (bones, connective tissue, nerves and muscles) and for diagnoses with impact on scoliosis (hormonal disorders, operations in the early childhood, etc.).

**Results**

Pathologies with a direct or indirect effect on the development or progression on scoliosis were found in nearly 19,3 % (n-55) of the patients. In 17 cases leg-length discrepancy >1 cm was the cause (2 after trauma of the growth plate, 1 fibrous dysplasia, 1 hypoplasia of the fibula, 1 hypoplasia of the femur, 2 chronic slipped capital femoris, 1 unknown Perthes disease could be found).In 10 cases a syndrome could be found (3 Marfan Sy., 1 Ehlers Danlos Sy., 2 Neurofibromatosis, 4 Prader Willi Sy., 1 Moebius Sy.). An operation in the early childhood was done in 4 cases (3 heart operations, 1 atresia of the esophagus). Malformations could be dedected in 7 patients (5 Hemisacralisation, 1 Patella Nail Syndrome, 1 M. pectoralis major hypoplasia). 4 patients showed a spondylolysis or developed a spondylolisthesis. Different tumor or tumor like lesions could be found in 3 cases (1 Medulloplastoma, 1 aplastic anemia, 1 eosinophilic granuloma). Hormonal disorders could be dedected in 7 cases (3 Hashimoto thyreoiditis, 1 Hypothyreoiditis, 3 growth hormone treatment). Also 1 case with a massive disc herniation, 1 CRMO and 1 case after meningitis in the early childhood could be dedected.

**Conclusion**

The compensation of leg length difference is the basic requirement before brace treatment.The problem of lumbosacral transition fault is still too little attention. The current MRI-technique is not sensitive enough to differentiate all causes. But the MRI-scan is necessary to detect different diagnosis in the spine.At every appointment history of the patient and her family should be part of the examination. Sometimes it takes years to find the right diagnosis. Especially to diagnose different syndromes can also be expensive.The improvement of the examination lowers the number of “idiopathic” scoliosis.

### O14 The effectiveness of core stabilization exercises versus conventional exercises in addition to brace wearing in patients with adolescent idiopathic acoliosis

#### Gozde Gur^1^, Necdet Sukru Altun^2^, Yavuz Yakut^1^

##### ^1^Hacettepe University, Çankaya, Ankara, Turkey; ^2^Akay Hospital, Ankara, Turkey

**Introduction**

Conservative treatment of Adolescent idiopathic scoliosis (AIS) involves a variety of physical exercises and bracing. For some of these treatments there is insufficient evidence. The aim of this study was to investigate the effects of core stabilization exercises versus conventional exercises in addition to brace wearing on trunk asymmetry, perception of deformity and health related quality of life in patients with AIS.

**Material and methods**

Nineteen female subjects with AIS were randomly placed into two treatment groups: Group 1 (*n* = 9, mean age 14,4 ± 2,1 years) core stabilization exercises (CSE) and brace; Group 2 (*n* = 10, mean age 14,1 ± 1,5 years) conventional exercises (CE) and brace. The average Cobb angle of the major curve was 32,2 ± 11,8° for thoracic (range: 14°–54°), 27,5 ± 6,9° for lumbar regions (range: 16°–34°) for the first group, 32,6 ± 7,2° for thoracic (range: 19°–40°), and 36,8 ± 7,9° for lumbar regions (range: 23°–50°) for the second group. The trunk asymmetry was assessed by Posterior Trunk Symmetry Index (POTSI), perception of deformity of physiotherapist and patient by Walter Reed Visual Assessment Scale (WRVAS), spinal rotation degree by scoliometer with Adam’s forward bend test, and health related quality of life by SRS-22. Measurements were carried out at baseline examination, following the completion of treatment. Exercises were performed one hour a day and seven days per week for ten weeks. Two sessions of exercise treatment were performed in the clinic by a physical therapist with three day intervals and between these intervals patients performed the same exercises at home. Patients were instructed to wear brace 23 hours each day. Results were analyzed using The Wilcoxon rank-sum test to compare repeated measurements and Mann-Whitney U test to compare two groups.

**Results**

Both thoracic and lumbar rotation was improved in CSE group (*p* < 0,05). In CE group, only lumbar rotation was improved after a ten-week treatment period. Also, improvement in lumbar rotation was better in CSE group (*p* < 0,05). Physiotherapists’ WRVAS total scores were better after treatment in both groups (*p* < 0,05). But there was no difference between groups. Patient’s WRVAS total scores did not change after treatment (*p* > 0,05). POTSI scores were improved in both group but there was no significant difference between groups (*p* > 0,05). There was no difference in both groups after treatment in terms of SRS-22 subgroups, self-image, mental health and total score except for function and pain in CSE group. Function and pain improved with CSE treatment.

**Conclusion**

We concluded that core stabilization exercises therapy improves trunk symmetry, spinal rotation and rib hump, function and pain in addition to brace treatment in patients with AIS. Further studies with more cases are needed to demonstrate effectiveness of this method in AIS conservative treatment.

### O15 The effect of physiotherapy techniques on the body balance in patients with scoliosis treated with corrective appliances

#### Piotr Gawda, Piotr Majcher

##### Uniwersytet Medyczny w Lublinie, Lublin, Poland

**Background**

The body vertical orientation in the upright standing position is maintained by keeping the body center of gravity (COG) upright above the base of support by a dynamic interplay of visual, vestibular, and somatosensory control systems. The relationship between balance control and independent mobility is particularly important in the population of individuals with severe scoliosis who have difficulties in balance maintenance and a decreased confidence in performance of daily activities. The control of upright stance can change during conditions of increased postural tension which limits the synergy of muscles system. Central nervous system receives a very large number of stimuli information coming from myofascial structures. The application of external forces that arise using corrective appliances leads to stimulation of mechanoreceptors of these structures, which affects the current balance control of the body. The force platform technique is one of the most frequently used quantitative techniques for postural control assessment that enables the measurement of the COG sway velocity . Objectives of this study are to point out the impact of the braces treatment on the postural control strategy in patients with scoliosis and to estimate the influence of the stretching therapy on the postural control strategy in patients with scoliosis treated with corrective appliances.

**Materials and methods**

The studied group consists of 24 girls between the ages of 10 to 15 years old. All diagnosed with idiopathic scoliosis and qualified to treat with corrective appliances. Twelve of them (group A) had been prepared for appliances corrections by rehabilitation with the use of stretching techniques, the rest (group B) without. Cobb angle of the respondents ranged from 25 degrees to 46 degrees. Postural characteristics of the subjects were measured with the use of a computerized force platform. The software program filters the center of pressure data and then calculates COG. The mCTSIB assesses a person’s ability to use sensory inputs for balance and distinguishes between normal and abnormal balance performance. This test measures COG sway velocity while standing in the different conditions. All evaluations were compared in two groups. All variables had normal distribution and were analyzed with parametric statistical tests. The differences of continuous variables among patients’ groups were determined with T test. The Fisher exact test was used to test the association for categorical data. Results with p-values <0.05 were regarded as statistically significant.

**Results**

The individuals with corrective appliances showed a significantly higher mean COG sway velocity as compared to the same patients before appliances. The increase in speed of COG deviation was positively correlated with the size of the angular correction of the curvature. Selected rehabilitation treatment with using stretching techniques reduces COG sway velocity increase after the appliance of the corrective braces.

**Conclusion**

Postural stability is decreased in patients with scoliosis just after applying the corrective braces. In order to maintain postural stability after the appliances a series of physiotherapy techniques**,** exercises of the antigravity muscles to prepare for the correction of soft tissue are necessary.

### O16 New combine method treating AIS – preliminary results

#### Lior Neuhaus Sulam

##### The Israeli Scoliosis Center, Tel Aviv, Israel

**Background**

The ApiFix is a novel non-fusion system of treatment for AIS. The system involves an expendable rod attached to the spine by only 2 screws, inserted around the apex of the major curve. The system is designed to gradually increase its length when the patient is doing exercises which increases the distance between the two screws considering the other curves. The goal of the system is to act as an “Internal Brace”, reduce the curve to below 35 degrees and maintain it at that level. The surgical procedure is significantly less invasive compared to the long fusion. The incision is around 10 cm, operation time around 1 hour and after 2-3 days the patient goes home.

40 patients were enrolled so far, 20 of them in controlled clinical trials in Europe.

**Aims**

To present a new method for treating AIS patients classified as Lenke 1 with a flexible curve, up to 60 degrees Cobb angle. The method comprises a small surgery combined with specific physiotherapy. Preliminary results of 9 cases.

**Design**

Retrospective case report of 9 girls treated along the past 2 year.

**Material and methods**

9 females, age 12.5-24, most passed growth spurt, with main Rt thoracic 41^0^-64^0^ cobb curve had Apifix operation with specific PT program 1 month before op and 6 month after the op including home exercise program. Assessment made by x-rays in standing and side flex, clinical assessment before op, and standing xrays with clinical assessment after the op, 2 weeks, 4 weeks, 3 m and 6 m.

**Results**

The improvement of the cobb was between 10 %-56 %, average of 31 % of correction, The improvement of the TRACE was 28 %-45 %, average 38 %, The improvement of the ATR was 9 %-42 %, average 27 %.

**Conclusion**

The main purpose of the this treatment is to stabilize and support the spine preventing progression in adult life without limiting the normal movement of the spine, decrees torsion forces, less muscle power against gravity, better cosmetics and less surgery complications. Reducing the Cobb under 35^0^, the TRACE and the ATR, will help the patient to achieve this aims.

It is important to follow the inclusions criteria to this procedure to have the best results. In addition it is crucial to start Physiotherapy treatment at least one month before op, the 2 girls in the bottom of the table which were the first operated in Israel start the PT treatment only after the op including the clinical assessment.

There is a need to have longer follow up to validate stable results in adulthood.

### O17 Does a 4-week intensive course of ScolioGold therapy reduce angle of trunk rotation in scoliotic patients: a retrospective case series

#### Michael Bradley^1^, David Glynn^2^, Alex Hughes^1^, Erika Maude^1^, Christine Pilcher^1^

##### ^1^Scoliosis SOS Clinic, London, Great Britain; ^2^University of York, York, Great Britain

**Background**

Angle of Trunk Rotation (ATR) affects rib and lumbar prominences, which can significantly influence a patient's back shape, and may negatively impact body image and self-esteem. Various studies have also linked ATR to Cobb angle for prediction of curve progression without the need for repeated radiation exposure from X-rays, with varying degrees of success. Therefore, ATR remains an important clinical outcome measure in practice.

The Bunnell Scoliometer is widely used as a basic method for measurement of ATR. ATR is the angle between the horizontal and the plane across the back at the greatest elevation of a rib prominence or lumbar prominence.

**Design and level of evidence**

The study was a retrospective case series in which 305 patients (47 males and 258 females) treated between December 2011 and June 2014 were measured for vertebral rotation by a ScolioGold Therapist using the Scoliometer in a standardised forward-bend position at the beginning and end of a 4-week intensive course of ScolioGold treatment.

Patients were aged between 8 and 76 years old (mean 26.6), and were only included if at least one curve had rotation > =5^o^ as measured by Scoliometer at start of treatment. There was no randomisation of patients.

**Material and methods**

Scoliometer readings were taken with each patient at the start and end of their 4-week ScolioGold treatment course. Each measurement was taken in a standardised seated forward-bend position, with knee height being consistent at both time points, and the same Scoliometer used. These results were then documented and anonymised before the values were analysed by an independent statistician. Paired t-tests were used to evaluate the difference between sum of ATR at start and end of treatment.

ATR is a reliable measurement with good reproducibility. Previous studies have suggested that the Scoliometer has excellent intra- and inter-observer agreement, with a change of 2 degrees reported previously to be clinically significant.

**Results**

In the cohort measured, average sum of total ATR reduced from 18.04 degrees (SD 6.70) to 14.30 degrees (SD = 6.15).

Single thoracic curvatures (*n* = 48): Mean reduction in ATR of 2.33 degrees (SD 0.42, *P* < 0.05).

Thoracolumbar curvatures (*n* = 54): Mean reduction in ATR of 3.05 degrees (SD 0.56, *P* < 0.05).

Double curvatures (n = 190): Mean reduction of ATR of 1.71 degrees (SD 0.25) and 1.95 degrees (SD 0.31) in thoracic and lumbar respectively (*P* < 0.05).

Post-spinal fusion (n = 13): Mean reduction of ATR of 4.19 degrees (SD 1.54, *P* < 0.05).

There is a statistically significant difference in the sum of ATR before and after treatment with the ScolioGold method.

**Conclusion**

ScolioGold therapy proved effective at reducing ATR magnitude for this case series to both a clinically and statistically significant degree in single thoracic or thoracolumbar curvatures. There was a statistically significant reduction of sum of ATR in double curvatures, but the literature is divided as to whether this change was clinically significant or not. These results were not statistically affected by whether treatment was completed as one 4-week block, or if they were split into two 2-week treatment blocks.

### O18 Schroth physiotherapy method without bracing is an effective treatment for scoliosis in improving curves and avoiding surgery and should be offered as a treatment option for scoliosis in Canada: case series

#### Andrea Lebel^1†^, Victoria Ashley Lebel^1,2†^, Judit Orbán^1^

##### ^1^Ottawa & District Physiotherapy Clinic, Scoliosis Physiotherapy and Posture Centre, McLeod Street, Ottawa, K2P 0Z8, Canada; ^2^Saba University School of Medicine, Saba, Dutch Caribbean, Netherlands

###### **Correspondence:** Andrea Lebel – Ottawa & District Physiotherapy Clinic, Scoliosis Physiotherapy and Posture Centre, McLeod Street, Ottawa, K2P 0Z8, Canada

^†^These authors contributed equally to this work

**Background**

Idiopathic scoliosis (IS) is a complex multifactorial three-dimensional (3D) spinal deformity of unknown cause. The treatment options offered for IS by orthopedic surgeons in Canada are observation for curve progression, bracing, and spinal fusion surgery. Physiotherapeutic scoliosis-specific exercises (PSSE) are currently not recommended by orthopaedic surgeons in Canada, even though it has been proven effective in preventing scoliosis curve progression and in a number of cases, reducing scoliosis curve angles (measured in Cobb degrees). Schroth physiotherapy can be effective in reducing the number of braces prescribed in Canada and in reducing the number of spinal fusion surgeries. The purpose of this study series is to evaluate the effect of an outpatient Schroth physiotherapy program in patients with IS and high risk of progression and who have not received bracing as treatment, by following primary curve degree Cobb angles and the angle of trunk rotation (ATR) measurements based on initial and follow-up radiographs and scoliometre measurments.

**Methods**

This retrospcetive case series includes 6 female patients ages 5-23 years diagnosed with IS. All study patients required an initial diagnostic radiograph, taken no earlier than 6 months before beginning Schroth physiotherapy, and a follow-up radiograph, taken within 6 months of completing Schroth physiotherapy. None of the case series patients received any form of bracing treatment prior to or during this study.

**Results**

In the period between initial diagnostic radiographs and follow-up radiographs, primary scoliosis curves showed an improvement of 10-19 degrees Cobb angle and ATR measurements improved by 2-8 degrees. Two years after the follow-up radiographs were taken, the ATR measurements of all 6 patients have remained stable. None of the patients required surgery.

**Conclusions**

Schroth physiotherapy without bracing is an effective treatment option for IS even in patients with a high risk of curve progression. A Schroth physiotherapy exercise program can improve scoliosis curve Cobb angles and ATR measurements, eliminating the need for surgery and/or bracing in a number of cases, as well as decrease the risk of scoliosis curve progression into adulthood.

### O19 Rotation of the trunk and pelvis and coupled movements in the sagittal plane in double support stance in adolescent girls with idiopathic scoliosis

#### Agnieszka Stępień, Krzysztof Graff

##### Józef Piłsudski University of Physical Education, Warsaw, Poland

**Introduction**

Scientists are still looking for causes of scoliosis and its progression. Trunk and pelvic movements in the transverse plane were evaluated only in a few studies [1,2]. A few authors indicated gait pattern as a reason of scoliosis progression [3].

**Aim**

The aim of this study was to determine the trunk and pelvis rotation range of motion (TR - trunk rotation, PR - pelvic rotation) in adolescent girls with idiopathic scoliosis (AIS) in a position imitating the double-support phase (DSP) of gait. The additional aim was to describe angular motions in the sagittal plane (MSP) occurring during rotation.

**Methods**

59 AIS girls (age 10-18, average 14,4) with the right thoracic curve or/ and the left lumbar curve were subsequently qualified to the study. Four groups including girls with different types of scoliosis were formed. Measurements were taken in the standing position imitating DSP. A special designed prototype axial rotation tester with the computer system was used to assess TR and PR and coupled MSP. The shoulder girdle with upper part of the trunk was stabilized during pelvic movements. The pelvis was fixed during trunk rotation motions. Special sensors were used to control feet motions. The number and order of motions were precisely determined. Right TR in the position with the right lower limb in front was compared to left TR in the position with the left lower limb in front. Right PR in the position with the left lower limb in front was compared with left PR with the right lower limb in front. 30 healthy girls without scoliosis were tested as the control group. ANOVA test and T-test were used for statistical analyzes.

**Results**

Significant difference between the right and left TR was found in girls with double curve scoliosis with the dominant thoracic curve. Left TR was significant lower than TR to the right. Differences between the right and left PR were not observed in groups.

PR to the right and left in girls with the lumbar curve was significant larger than in other groups. TR was coupled with characteristic MSP. Left TR was coupled with forward trunk movement and right TR was associated with trunk backward movement in the majority of participants.

The posterior pelvic tilt was observed during the left PR in girls with the lumbar curve. The increased anterior pelvic tilt appeared in girls with the single thoracic curve during the right PR.

**Conclusions**

TR / PR values and coupled MSP depend on a scoliosis type and direction of rotation. It is important to pay attention during physiotherapy to coupled spine movements which occur during rotation. Observed differences can be one of causes of gait pattern asymmetry in AIS. This hypothesis needs confirmation.

**References**

1. McIntire Kevin L, Asher Marc A, Burton Douglas C, Liu Wen. Trunk rotational strength asymmetry in adolescents with idiopathic scoliosis: an observational study. Scoliosis. 2007; 2: 9.

2. Stępień A. A range of rotation of the trunk and pelvis in girls with idiopathic scoliosis. Advences in Rehabilitation 2011, (3), 5-12. (article in Polish)

3. Burwell RG, Cole AA, Cook TA, Grivas TB, Kiel AW, Moulton A, Thirwall AS, Upadhay SS, Webb JK, Wemyss-Holden SA, Whitwell DJ, Wojcik AS, Wythers DJ: Pathogenesis of idiopathic scoliosis. The Nottingham concept. Acta Orthop Belg 1992, 58:33-58.

### O20 Curve progression analysis in Risser 0 patients orthotically managed with compliance monitors

#### D. Speers

##### Scheck and Siress, Chicago, IL, USA

**Background**

Bracing for adolescent idiopathic scoliosis is the major modality for conservative care [1]. Recognizing, and treating, scoliosis at a young age has shown to be beneficial for successfully, conservatively managing scoliosis and not letting it progress to surgical levels. Increased dosing has shown to proportionally correlate to success levels with bracing.

**Aim**

To see if curves remained stable or decreased in the Risser 0 population while undergoing brace treatment in out of brace x-rays (72 hours out of brace).

**Methods**

Eight children with idiopathic scoliosis and Risser 0 maturity managed with a tlso had out of brace x-rays taken (72 hrs out of brace) to document curve status. The curves were categorized as *increased* (greater than 5 degrees progression), *within error* (within 5 degrees above or below initial curve measurement), or *decreased* (greater than 5 degrees regression). Average time in brace 14.01 hours.

**Results**

For the eight Risser 0 patients, two had curves that decreased, four were within error, one increased and one is to have out of brace x-rays within the next month.

**Conclusion**

Bracing the Risser 0 population appears to slow progression and even reverse scoliotic curves.

**References:**

1. Effects of bracing in adolescents with idiopathic scoliosis. Weinstein SL, Dolan LA, Wright JG, Dobbs MB.

2. Brace wear control of curve progression in adolescent idiopathic scoliosis. Katz DE, Herring JA, Browne RH, Kelly DM, Birch JG.

3. Validation of a miniature thermochron for monitoring thoracolumbosacral orthosis wear time. Benish BM, Smith KJ, Schwartz MH.

### O21 Conservative treatment in Scheuermann's kyphosis: comparison between lateral curve and variation of the vertebral geometry

#### Angelo Gabriele Aulisa^1^, Vincenzo Guzzanti^1^, Giuseppe Mastantuoni^1^, Andrea Poscia^2^, Lorenzo Aulisa^3^

##### ^1^U.O.C. of Orthopedics and Traumatology, Children's Hospital Bambino Gesù, Institute of Scientific Research, P.zza S. Onofrio 4, Rome, Italy; ^2^Institute of public health, University Hospital "Agostino Gemelli", Catholic University of the Sacred Heart School of Medicine, Rome, Italy; ^3^Department of Orthopedics, University Hospital "Agostino Gemelli", Catholic University of the Sacred Heart School of Medicine, Rome, Italy.

**Background**

Conservative treatment of vertebral deformity promotes with the application, of external forces to obtain, via appropriate geometry orthosis, during skeletal growth, remodelling of the deformed vertebras. In a previous paper on Scheuermann's kyphosis, we have studied the geometry variations of all vertebrae included in the curve, before and after the treatment.

**Design**

The purpose of this prospective study was to confirm the effectiveness of conservative treatment in Scheuermann's kyphosis and was to evaluate and compare the variation of the vertebral geometry with the curve trend in Cobb degrees, before and after conservative treatment.

**Material and Methods**

This prospective study was conducted on 90 patients with thoracic Scheuermann's kyphosis, treated using anti-gravity brace: 59 male, 31 female. The mean age at the beginning of the treatment was 14 years.

Radiographical measurements were performed on radiographs from a lateral projection, at the beginning (t1) and at the end of the treatment (t5).

To avoid the great variance in the range of curve angles in thoracic kyphosis that rely on the radiological position, x-rays were performed all at our Radiology Department observing the following position: standing with head straight, arms bent at 45° and hands placed on a support.

Vertebral geometry modifications at t1 and t5 were analysed according to the following parameters and evaluated by three independent observers:

Anterior wedging angle (ALFA) of the apex vertebra and Posterior wall inclination (APOS) of the limiting lower vertebra. These parameters were chosen because they had shown to be the most significant in a previous study.

The curve was measured in Cobb degrees.

Statistical analyses was performed.

**Results**

The results from our study showed that of the 90 patients with a thoracic curve mean value of Cobb degrees was 57.8 ± 6.0 SD at t1 and 41.3 ± 5.6 SD at t5. The differences between t1(angle at baseline) and t5 (end of treatment) were calculated for Cobb, alpha and Apos angle and were respectively -16.4 ± 4.5, -6.4 ± 1.4 and -2.7 ± 1.2; tested with paired t-test were significative (*p* < 0.01). The results of the regression analysis to test the relationship between the three measures for the kyphosis (cobb degree, alpha and Apos) showed that the best association was between Cobb t5 and Alpha t5 (*p* < 0.01) and between Cobb t1 and Apos t1 (*p* < 0.01). No significative association was found between the difference between alpha and Apos.

**Conclusion**

Our results confirm that conservative treatment in Scheuermann's kyphosis can remodelling the deformed vertebras.

We sustain that using new parameters to study vertebral remodelling allows us to reach a better comprehension of Scheuermann spine response to anti-gravity brace treatment.

Furthermore, the evaluation of the alpha angle of the apex vertebra confirms to be more reliable than Cobb’s angle because it cannot be affected by the radiological position

### O22 The plaster cast in the conservative treatment of idiopathic scoliosis can still play a positive role?

#### Angelo Gabriele Aulisa^1^, Vincenzo Guzzanti^2^, Francesco Falciglia^1^, Andrea Poscia^3^, Lorenzo Aulisa^4^

##### ^1^U.O.C. of Orthopedics and Traumatology, Children's Hospital Bambino Gesù, Institute of Scientific Research, P.zza S. Onofrio 4, Rome, Italy; ^2^U.O.C. of Orthopedics and Traumatology, Children's Hospital Bambino Gesù, Institute of Scientific Research, P.zza S. Onofrio 4, Rome, Italy; University of Cassino, Cassino, Italy; ^3^Institute of public health, University Hospital "Agostino Gemelli", Catholic University of the Sacred Heart School of Medicine, Rome, Italy; ^4^Department of Orthopedics, University Hospital "Agostino Gemelli", Catholic University of the Sacred Heart School of Medicine, Rome, Italy

**Background**

The current treatment of idiopathic scoliosis is based on the development of protocols and guidelines that show the way and the time needed until the conservative treatment results effective. The first of these protocols included the use of three corrective plaster casts before applying the brace. However, in the last years, many schools have abandoned the plaster cast both to increase compliance and because convinced of the effectiveness of the brace.

**Design**

The purpose of the present study was to evaluate whether the corrective plaster cast positively affects on the outcome and if its use can still play a positive role in the conservative treatment of scoliosis.

**Material and methods**

From a consecutive series of patients, included in a prospective database, to whom had been proposed the indication for a corrective plaster cast, to improve the effectiveness of conservative treatment, 128 scoliosis were selected: 78 thoracic (12.6 ± 1.8 years) treated with Lyon brace and 50 lumbar or thoracolumbar (12.9 ± 1.8 years) treated with PASB. The 50 % of patients had accepted the treatment with plaster cast before the application of the brace. In these cases 2 corrective plaster casts for about 20 days each were applied.

X-rays were used to estimate the curve magnitude (CM) and the torsion of the apical vertebra (TA) at 3 time points: beginning of treatment (t1), four months after the beginning (t2), 2-year minimum follow-up (t3). Three outcomes were distinguished in agreement with SRS criteria: curve correction, curve stabilization and curve progression.

**Results**

The results from our study showed that of the 78 patients with a thoracic curve CM mean value was 40.9 ± 7.1 SD at t1 and 27.1 ± 9.4 SD at t3. The 50 patients with a lumbar curve CM mean value was 39.4 ± 6.8 SD at t1 and 23.3 ± 9.3 SD at t3. The difference in cobb degrees between t3-t1 was 16.5° in thoracic curves treated with plaster cast and 11.2° in those treated without plaster cast while in lumbar curves was respectively 19.7° and 12.5°.Therefore the patients who used the plaster cast before starting treatment with brace achieved an higher improvement in cobb degrees than those who have not used it (5.71 cobb degrees, *P* < 0.01).

The patients with higher Cobb degree at baseline showed a better evolution of the scoliosis at T2 if they used the plaster cast (-22.4° vs -11.6°; *p* < 0.01), even if at t3 they showed a little worsening (-17.5° vs -12°; *p* < 0.01). Similarly, even patients with lower Cobb degree at baseline showed a better evolution of the scoliosis at t2 if they used the plaster cast (-19.9° vs -9.0°; p < 0.01), with a little worsening at t3 (-17.5° vs -11.8°; *p* < 0.01).

Curve correction was accomplished in 114 patients (89.1 %), stabilization in 10 patients (7.8 %). 4 patients (3.1 %) had a curve progression.

**Conclusion**

In all cases, the treatment with corrective plaster cast showed a better outcome compared to the non-plaster, even in the lower curves. Therefore, considering the results in greater curves, to ensure a better QOL, the treatment with plaster cast should be the first choice.

While in the lower curves, to improve compliance, no indication to the plaster cast can be given even if outcomes are better.

### O23 Bracing for Adolescent Idiopathic Scoliosis (AIS) and Scheuermann Kyphosis : The issue of overtreatment in Greece

#### Nikos Karavidas

##### Scoliosis Spine Laser Centre, Athens, Greece

**Introduction**

Most recent evidence has proved the efficacy of brace in the treatment of spinal deformities for young adolescents. Scoliosis Research Society (SRS) and Society on Scoliosis Orthopedic Treatment (SOSORT) have produced guidelines to indicate when brace treatment must be applied. The purpose of this study was to evaluate the rate of overtreatment for AIS and Kyphosis in Greece, according to SOSORT and SRS guidelines. To date, this is the first study to investigate overtreatment percentage in a group of patients with spinal deformities.

**Material and methods**

Cross-sectional study design and data analysis were performed in a group of patients that received treatment in a private clinic, in 2014. Of 289 treated patients, 167 young adolescents (128 females - 41 males, mean age 15, 7 years) were eligible for inclusion criteria (age 9-18 years, brace wearing). Overtreatment was defined as the unnecessary use of brace according to SRS and SOSORT indications for brace treatment, and referred to individuals that should have never started wearing a brace or to those that brace weaning was very prolonged. Overtreatment was assessed by a Schroth certified Physiotherapist, by estimating Cobb angle, Risser sign, age of menarche in girls, and vertebral wedging in Scheuermann’s kyphosis, alongside with a subsequent analysis of risk prognostic factors (family history, angle trunk rotation, thoracic hypokyphosis, and curve type). The braces were prescribed by 34 medical doctors (MD) from different geographical areas of Greece.

**Results**

The data analysis revealed that 71 out of 167 subjects (42,5 %) had received some kind of overtreatment. The percentage of overtreatment was similar for AIS (51/118 patients, 43,2 %) and kyphosis (20/ 49 patients, 40,8 %). A further analysis showed that in the AIS subgroup, 20 subjects (16,9 %) had Cobb angle < 20^o^ , 7 subjects (5,9 %) had Cobb angle 20^o^ – 25^o^ but good prognosis, 12 subjects (10,2 %) started bracing after Risser 4 or 5, and 12 subjects (10,2 %) had not reached brace weaning even a long time after skeletal maturity. It is noticeable that 8 subjects (6,8 %) were at Risser 5 with Cobb angle < 20^o^ and were prescribed a brace. In the Kyphosis subgroup, 11 subjects (22,5 %) showed no signs of Scheuermann’s disease and no clinical rigidity, 3 subjects (6,1 %) started bracing after Risser 4 or 5, and 6 subjects (12,2 %) should have reached brace weaning much earlier.

**Conclusions**

An extremely high rate of overtreatment (42,5 %) was identified in a random group of adolescents treated with a brace for AIS and Kyphosis. This is probably attributed to lack of knowledge in the field of conservative treatment of spinal deformities. It also seems that the majority of MDs ignore the role of the Physiotherapeutic Scoliosis Specific Exercises (PSSE) in the treatment of scoliosis. Overtreating a child with a brace can cause social, financial and psychological problems to them. The present study pinpoints the need for an evidence-based approach to conservative treatment of idiopathic scoliosis and kyphosis, according to SOSORT and SRS guidelines, in order to avoid overtreatment and to enhance clinical outcomes.

### O24 Efficacy of Milwaukee brace for correction of scheurmann kyphosis

#### Mohammadreza Etemadifar

##### Orthopedic Department, Esfahan University of Medical Sciences, Isfahan, Iran

**Introduction**

Scheurmann's kyphosis is a relatively common sagittal plane mal alignment inadolescents, which could be corrected relatively easily provided that is diagnosed earlyand treated with brace. There are several kinds of braces useful in treatment of thisdeformity in which Milwaukee is the most popular one. Few studies recently havedemonstrated brace efficacy in treating scheurmann kyphosis. The aim of our study wasto investigate effects of a low-profile Milwaukee brace for treatment of this deformity.

**Material and methods**

All adolescent patients with diagnosis of scheurmann kyphosis with at least one year ofgrowth remaining were included in our study. Standing AP/LAT (T1- S1) X-rays wasperformed in all cases. Thoracic kyphosis Cobb angle and vertebral wedging, end platesclerosis and irregularity were identified and recorded. A modified low profile Milwaukee brace (without neck ring) was administered for all patients. Follow up X-rayswere performed at 2, 6 and12 months of brace wear and one year after brace weaning. T-test was used to compare average pre and post- brace kyphosis.

**Results**

A total of 158 patients were enrolled in our study. 32 cases were lost at final follow up (including 5 cases who were shifted to surgery). 126 cases were remained for evaluation.Average age of the patients was 13.5 years (10 to 16 years). There were 78 females and 48 males. Average primary thoracic kyphosis was 67 degrees and after 2, 6 and 12 months brace wear it was 50, 41 and 33 degrees respectively. Average full time bracewear was 18 months and at the last follow up mean kyphosis angle was 45 degrees,which was statistically improved compared to primary kyphosis (*p* < 0.05). All patientsand parents were satisfied with results.

**Conclusion**

Low profile Milwaukee brace can be effective for conservative treatment of scheurmann kyphosis provided that it is well fitted and used regularly by the patient. Although there issome correction loss, final thoracic kyphosis is acceptable and most patients are satisfied with results.

### O25 The three dimensional analysis of the Sforzesco brace correction

#### Sabrina Donzelli^1^, Fabio Zaina^1^, Monia Lusini^1^, Salvatore Minnella^1^, Luca Balzarini^2^, Stefano Respizzi^2^, Stefano Negrini^3,4^

##### ^1^ISICO Italian Scientific Spine Institute, Milan, Italy; ^2^ ICH Istituto Clinico Humanitas, Milan, Italy; ^3^IRCCS Don Gnocchi, Milan, Italy; ^4^University of Brescia, Brescia, Italy

**Background**

Scoliosis is a three dimensional deformity, and brace correction should be 3D too. There is a lack of knowledge of the effect of braces, particularly in the sagittal and transverse plane. The aim of this study is to analyse the Sforzesco Brace correction, through all the parameters provided by Eos 3D imaging system.

**Method**

*Design:* This is a cross sectional study from a prospective database started in March 2003.

*Participants:* 16 AIS girls (mean age 14.01 ) in Sforzesco brace treatment, with EOS x-rays , at start, in brace after 1 month and out of brace at 4 months. *Outcome measures: All the parameters and the Torsio-Index obtained from 3D Eos System, in and out of brace, in the three planes.* Statistical analysis: *the variability of the parameters and the mean differences were analysed and compared using paired T test. ANOVA was used for multiple comparisons. P value was set below 0.05.*

**Results**

In the comparison in brace vs start of treatment the mean Cobb angle change significantly from 36.44 + -4 to 28.99 + -3.9 (*p* = 0.01). Significant changes in all the sagittal parameters were found (*p* = 0.02). In the axial plane, the TorsioIndex, changed significantly in brace, only for thoracolumbar and lumbar curves (*P* < 0.05). The analysis of the single vertebral tilt, demonstrated that the effect of brace are mostly concentrated to some segments:T4-T5; T10-T12, L1 and L5 in the AXIAL plane and T3-T6; T10-L1 in the frontal plane.

**Conclusion**

Sforzesco brace mostly modify the middle of the spine, and preserve the sagittal balance. The single vertebral orientation in each plane, should be considered together with the typically used values to assess brace effect.

### O26 Quality of Life in adolescents with idiopathic scoliosis: A comparison measured by the Kidscreen 27 between scoliotic patients and healthy controls

#### Kathrin Güttinger

##### Zürcher Hochschule für angewandte Wissenschaften (ZHAW), Winterthur, Switzerland

**Background**

Different questionnaires evaluate the quality of life in scoliotic patients. The questionnairs which were developed especially for scoliotic patients are: Brace Questionnaire (BrQ) , Bad Sobernheimer Stress Questionaire Brace and Deformity (BSSQ-Brace/ BSSQ-Deformity), SRS-22 and Scoliosis Quality of Life Index (SQLI).

In addition there are the questionnaires which measure the general quality of life in children and adolescents: SF 36, Kidscreen 52 & 27.

**Aim**

The aim is an evaluation of the quality of life and the self-efficacy of adolescents with idiopathic scoliosis (IS). The goal of the study is to compare the results of the adolescents with scoliosis with the results of the adolescents without scoliosis.

**Design**

It is a cross-sectional design study. 30 scoliotic patients and 30 healthy adolescents participated in the study. The inclusion criteria for the patients group was: IS, age between 12 & 18 years, girls and Cobbangle > 25°. The inclusion criteria for the control group was: girls, age between 12 & 18 years and no known scoliosis.

**Material & methods**

30 patients were included (mean age 14. 4 years, mean Cobbangle 36.1 °) and 30 healthy controls (mean age 14.8 years). Both groups filled out the kidscreen 27 and special additional questions. The additional questions were:

1. the subgroup About yourself of the kidscreen 52,

2. special questions formulated by the author,

3. questions about the self-efficacy, also formulated by the author and

4. questions about the sport activities and the school type (both groups) and the brace wearing time and the therapy intensity (in the patientsgroup).

The Kidscreen 27 consists of 5 categories: Physical Well-Being (5 items), Psychological Well-being (7 items), Autonomy & Parents (7 items), Peers & Social Support (4items) and School Environment (4 items). The higher the score the better the quality of life. To make the score generally comparabel, the score was transfered with an Raschanalysis.

**Results**

In the dimension Psychological Well-Being the scoliotic patients had higher T-Scores than the control (48,9 +/- 7,42 versus 46,13 +/-8,40). Also in the categorie About yourself the patients had higher scores (47,65 +/-7.64 versus 43.4 +/-6.23).

In the categorie Autonomy and Parents the patient group had lower T-Scores than the controls (52.09 +/- 6.96 versus 55.03 +/-8.95). This is the same for the category Peers and Social Support (51.1 +/- 7.7 versus 53.06 +/- 7.92). But in the comparison these two categories between the scoliosis patients and the standard value of switzerland the scoliosis patients are not below the standard value (52, 51).

**Conclusion**

Adolescents with scoliosis showed better scores in the categorie Psychological Well-Being and About yourself than the controls. In the cateories Autonomy & Parents and Peers and Social they had lower scores.

### O27 The degree of illness acceptance in young women with idiopathic scoliosis treated with orthopedic braces: a preliminary study

#### Jacek Durmała ^1^, Irmina Blicharska ^1^, Agnieszka Drosdzol–Cop ^2^, Violetta Skrzypulec–Plinta ^2^

##### ^1^School of Health Sciences in Katowice, Medical University of Silesia, Chair and Department of Rehabilitation, Katowice, Poland, ^2^School of Health Sciences in Katowice, Medical University of Silesia, Chair of Woman’s Health, Katowice, Poland

**Background**

The degree of illness acceptance is one of the factors that have an impact on the patient’s quality of life. At present, this topic is frequently discussed due to ideological transformations in medicine, where the comprehensive approach towards the patient’s heath is taken into consideration. In the holistic model of medical care, the subjective dimension of ailment is very significant. The acceptance, however, has an influence on the self-esteem and it determines particular attitude towards the therapy.People with a higher degree of acceptance show better adaptation and lower intensification of negative emotions.

**Material and methods**

36 women (aged 20.7 ± 1.89 and diagnosed with adolescent idiopathic scoliosis) were qualified for the study. An average value of the Cobb’s angle in the primary curvature was 31.2° ± 12.39°. Patients were treated by means of kinesotherapy (within the range of DoboMed technique) in connection with the Chaneau brace. For the acceptance evaluation,the Acceptance of Illness Scale (AIS) was used. It is a tool developed by B. J. Felton and his colleagues (in the Polish adaptation by Zygfryd Juczyński). The scale contains eight statements (measured in points from 1-5), which describe negative consequences of poor health condition. The degree of acceptance is calculated by the amount of points within the range of 8 to 40. A low result indicates non-acceptance and ailment accommodation as well as strong feeling of psychological discomfort. A high score attests to the self-acceptance. The apical vertebral rotation (AVR) was determined on the basis of a current radiograph. The degree of trunk deformations was based on the topography of a surface of the body and the Posterior Trunk Symmetry Index (POTSI).The aim of the prospective research with randomization, conducted by means of double blind testing, was an evaluation of acceptance in women treated with scoliosis braces. The obtained results were analyzed by means of Statistica v.10. The assessment of variables concurrence with the typical layout was conducted with the test invented by Shapiro-Wilk. The dependence between parameters was determined on the basis of the Pearson’s R Correlation Test. The value p <0.05 was determined as a level of statistical significance.

**Results**

An average amount of points obtained from the Acceptance of Illness Scale was 33.1 ± 6.63. This indicates a high acceptance levelin a group of tested women with scoliosis. The considerable dependence between: (1) the amount of points in AIS, (2) the Cobb angle primary scoliosis (R = -0.3, p = 0.06), as well as (3) the AVR amount (R = -0.2, p = 0.24), hasn’t been observed. Statistically considerable correlation was determined between the degree of acceptance and the POTSI index (R = -0.4, p = 0.02). Women with a lower degree of trunk deformations present a higher level of scoliosis acceptance.

**Conclusions**

Women, diagnosed with scoliosis, indicate a high degree of its acceptance. Factors, which may determine the perception of a particular disease, are mainly trunk deformations and distortions. An angular curvature value does not considerably affect the level of scoliosis acceptance. It can be correlated with the lack of an accurate correspondence between the Cobb angle and trunk deformations in the coronal plane. The investigation requires further examination and supplementation.

### O28 Which are the personality traits of the patients with Adolescent Idiopathic Scoliosis?

#### Elisabetta D'Agata^1^, Judith Sánchez-Raya^2^

##### ^1^Vall d'Hebron Hospital Institute, Barcelona, Spain, ^2^Vall d'Hebron Hospital, Barcelona, Spain

**Background**

According to the bio-socio-psychological model, for a more complex understanding of the patient, biological aspects have to be integrated with psychological dimensions. This field in scoliosis is now emerging.

This study is about the main traits of personality in patients with Adolescent Idiopathic Scoliosis (AIS).

**Material and methods**

27 patients with AIS (aged 14.6 years; Mean Cobb Angle: 31°.5; 40.7 % braced) answered a Socio-Clinical Questionnaire, a Quality of Life tool (SRS-22), the Trunks Apperception Scale (TAPS) and a Personality Questionnaire (16PF- Adolescent Personality Questionnaire). 16 PF-APQ Questionnaire presents 16 scales and identifies 5 global dimensions.

**Results**

Results for SRS-22 subscales were: Function = 5; Pain = 4; Self Image = 3.6; Mental Health = 3.5. In 16 PF-APQ Personality Questionnaire, the personality scales with a percentile value > 50 were: Dominance (72 PCTL), Rule consciousness (63° PCTL), Vigilance (65 PCTL), Privateness (61 PCTL) Openness to change (60 PCTL) and Self- reliance (70 PCTL). With reference to the global dimensions, Independency corresponded to percentile 65 while Extraversion to 28.4. Neither the scales nor the dimensions presented any extreme percentiles. School note mean was 7.5.

Rule-consciousness related significantly (*p* < 0.001) to SRS-pain (Spearman r = 0.5) and to SRS-Self Image (Spearman r = 0.7).

**Conclusion**

Patients with Idiopathic Scoliosis did not present any psycho-pathological features. They appeared as introverted people with a tendency to be independent and assertive and with a good scholar success.

### O29 How many Scolioses do exist in the same person? A zoom vision on the perception of the patient

#### Judith Sánchez-Raya^1^, Elisabetta D'Agata^2^

##### ^1^Vall d'Hebron Hospital, Barcelona, Spain; ^2^Research Institut Vall d'Hebron Hospital, Barcelona, Spain

**Introduction**

The Trunk Appearance Perception Scale (TAPS) is a valid instrument for evaluating the perception patients have of their trunk deformity. There are no studies about the correlation among TAPS scored in each case by a physician, a patient and his /her parents. The object of the study is to compare the different perceptions of scoliosis and how the patient perception affects his/her quality of life.

**Material and methods**

The sample consisted of 64 patients (51females), mean age 15.25, mean Cobb Angle 30.6 (ranging from10°- 55°). 29 were not treated, 26 braced, 6 treated with physiotherapy. For each case, TAPS was scored individually by patient, his/her parents and the same doctor. Patients also scored Quality of Life Questionnaire (SRS-22).

The sample was split into two groups according to the age (1^st^ group: 9-14; 2^nd^ group: 15-34). Spearman correlation Index was calculated for the three TAPS, Cobb Angle and SRS-22 4 subscales (Function, Pain, Self Image, Mental Health).

**Results**

Correlations between parents’ TAPS and doctor’s TAPS was r = 0.5 (p < 0.001). Parents’ TAPS and doctor’s TAPS were statistically different, regarding the younger patients (Wilcoxon Signed Ranked Test, *p* = 0.02); besides, with relation to the older group, the correlation between parents and doctor TAPS was low (r = 0.4, *p* < 0.05). Correlations between patients and their parents as well as between patients and the doctor were moderate (r < 0.5).

The correlation between patient TAPS and SRS-22 Self Image had a moderate value in the younger group (r = 0.5) and a low one in the older group (r = 0.4, *p* < 0.05). The correlation between Body Image and Mental health was significative only for the younger subjects (r = 0.4, *p* < 0.005).

**Conclusion**

Doctor’s and parents’ perceptions are a bit discordant: in the younger group they are different, while in the older one their relation is moderate. Besides, in a patient the relation between his/her trunk perception with Body Image was moderate and associated to his/her age.

## ORAL POSTER PRESENTATIONS

### P1 The algorithm for the automatic detection of the pelvic obliquity based on analysis of the PA viev of the x-ray image

#### Sławomir Paśko^1^, Wojciech Glinkowski^2^

##### ^1^Warsaw University of Technology, Institute of Micromechanics and Photonics, Warsaw, Poland; ^2^Chair and Department of Orthopedics and Traumatology of the Locomotor System, Baby Jesus Clinical Hospital, Center of Excellence “TeleOrto” for Telediagnostics and Treatment of Disorders and Injuries of the Locomotor System, Medical University of Warsaw, Warsaw, Poland

**Background Information**

The oblique position of the pelvis frequently coincides with scoliosis. Asymmetrical location iliac crests may result in unequal pressure distribution while sitting and pain. It may reduce the patient’s tolerance of sitting position. The disease affects on knees, hip joints, feet and spine and can contribute to the emergence of diseases in these organs. Smaller degrees of the pelvic obliquity are almost to be accepted. It requires observation over the time; The problem may require relief before it becomes a greater clinical problem. The physicians can diagnose the problem of the pelvic obliquity based upon the analysis of PA view of the X-ray image. It is detected as the angular difference between the inter iliac crest line and the horizontal line. The value of this angle reflects the severity of the pelvis obliquity.

**Purpose**

The algorithm is intended to aid the automatic process of analyzing an X-ray image taken in PA view. It allows detecting horizontal positions of the upper edge of iliac crests. If the calculated positions are different then the physician is noted by the system of the possibility of occurrence of the pelvic obliquity. Greater degrees of the pelvic obliquity are easy to notice from the analysis of the X-ray image. Smaller degrees are difficult recognize without a referential horizontal line drawn on the image in the proximity of the pelvic bones. This process can be improved by the proposed algorithm that makes possible the detection of the small difference between the pelvic bones.

**Methods**

The multilevel algorithm processes an X-ray image based on the image processing. On the first level, the area of the pelvic girdle is roughly delimited. Due to this the upper part of the trunk is omitted from further analysis. Next they are designated few candidate areas in which pixels distribution may indicate that in one of them is located the searched edge of the pelvic bones. During the following level of processing, this set is narrowed to the maximum four candidates down. Finally, the last part of the algorithm is started where the best candidate chosen.

**Result**

The study was done on a collection of X-ray from scoliotic patients with or without the incidence of the pelvic obliquity. The set of images consisted of 10 digital PA view X-rays. The algorithm run for each image and results were saved. The same set was analyzed by specialist for the expert opinion. Positive matches were noted in analyzed cases.

**Conclusions and Discussion**

Algorithm based supported diagnosis can simplify the process of an X-ray image analysis and improve the reliability of this procedure. The physician can remain focused on the spinal curvature without the risk to skip minor radiologic signs on the radiographic image including the oblique position of the pelvis. The bigger radiographic collection should be tested for more extensive research of the algorithm to calculate its reliability.

Acknowledgement:

This research was supported by project NR13-0109-10/2010, founded by National Center for Research and Development.

### P2 Monitoring of spine curvatures and posture during pregnancy using surface topography – case study and method assessment

#### Jakub Michoński^1^, Katarzyna Walesiak^2^, Anna Pakuła^1^, Robert Sitnik^1^, Wojciech Glinkowski^2^

##### ^1^Warsaw University of Technology, Warsaw, Poland; ^2^Department of Orthopaedics and Traumology of Locomotor System, Center of Excellence ”TeleOrto”, Medical University of Warsaw, Warsaw, Poland

**Background**

Lower back pain during pregnancy is a well-known problem. Ostgaard et al. report that almost 50 % pregnant women suffer from back pain [1]. Gutke et al. find that the frequency of lower back pain in pregnant women can be up to 4 times higher than in non-pregnant women [2]. In the prospective study of Kristiansson et al. 30 % women with the highest pain score report great difficulties with normal activities [3]. Moore at al. performed a study on the postural changes in pregnant women in 1989 [4], however did not use surface topography to achieve this goal.

**Design and level of Evidence**

A pilot study was conducted to test the potential of monitoring the change of spine curvatures and posture during pregnancy using surface topography. A single case was studied to test the methodology and preliminarily assess the usefulness of the procedure before performing a randomized trial. The apparatus used in this study was metrologically tested and utilized in scoliosis screening.

**Material and Methods**

The subject was measured using a custom-made structured light illumination scanner with accuracy of 0.2 mm. Measurement was taken every 2 weeks, between 17^th^ and 37^th^ week of pregnancy, 11 measurements in total. The subject was 34 years old at the beginning of the study, no systemic disorder, no drug use, no previous trauma or surgery of spine or lower limbs. From the measurement the thoracic kyphosis and lumbar lordosis angles, and vertical balance were extracted automatically. All measurements were repeated three times and the median calculated values were chosen. Oswestry Low Back Pain Disability Questionnaire (ODI) was done with every measurement.

**Results**

The values were correctly extracted from the measurement without any user interaction. From the registered values mean and standard deviation were calculated. The registered change was 4 degrees in kyphosis angle, 5 degrees in lordosis angle and 4 degrees in vertical balance angle. The calculated ODI index was between moderate disability and crippling back pain (20 % to 73 %).

**Conclusions**

We have found that surface topography is suitable for monitoring of spinal curvature and posture change in pregnant women. Automatic calculation of curvature angles and extraction of vertical balance angle allows to obtain high reliability of such a study without the observer errors introduced otherwise. No concrete conclusions can be drawn from the study because of insufficient amount of data, however flattening of lordosis in the evaluated case matches the conclusions of Moore et al.

**References**

1. Prevalence of Back Pain in Pregnancy. OSTGAARD, H C MD; ANDERSSON, G B J MD, PhD; KARLSSON, K MD

2. Predicting persistent pregnancy-related low back pain. A. Gutke, H.C. Ostgaard, B. Oberg. Spine, 33 (12) (2008), pp. E386–E393

3. Back Pain During Pregnancy: A Prospective Study. Kristiansson, Per MD*†; Svärdsudd, Kurt MD, PhD*; von Schoultz, Bo MD, PhD‡

4. Postural changes associated with pregnancy and their relationship with low-back pain. K. Moore, Msca, G.A. Dumas, PhD, J.G. Reid, PhD

### P3 Spinal rotation under static and dynamic conditions: a prospective study comparing normative data vs. scoliosis

#### Helmut Diers

##### Research & Development, Schlangenbad, Germany

**Background**

Using SST (Spine & Surface Topography) can significantly reduce the amount of harmful radiation in scoliosis treatment and follow-up during therapy. The SST under dynamic condition shoes the body movement, body mechanics and the activity of the muscles. People with scoliosis show under dynamic conditions reduced mobility and asymmetric lateral rotation.

**Aim**

The improvement of the spinal rotation during walking on the treadmill should be verified and this additional information should be integrated in the therapy.

**Design**

Patients according to different age groups and gender were measured with static 4D (habitual standing) and dynamic 4D motion (walking on the treadmill). The age of the patients was between 25-50 years. 20 Patients were measured two times. On measurement was under static conditions and the second under dynamic conditions. The focus was on the parameter “vertebral rotation”.

**Methods**

SST uses surface topography imaging for 3D back scanning and techniques creating 3D models of the spine without exposing patients to any ionizing radiation. The spine reconstruction model has been used successfully for more than 20 years in evaluation and treatment of patients with spinal deformities such as scoliosis, lordosis and kyphosis. Dynamic spine analysis is using similar algorithms as used in static measurement.

**Results**

Based on the degree of scoliosis there is a significant improvement of the vertebral rotation during walking on the treadmill. The rotation becomes more equivalent. In this study the parameter of the “vertebral rotation” was picked out and the differences between static and dynamic were worked out to get the output for the therapy.

**Conclusion**

The information of the spinal rotation is important for the therapy of patients with scoliosis. Regular monitoring of patients with scoliosis is under static conditions. The additional information of the dynamic and functional results provides important information towards personalized care and the organization of the therapy. The therapy should be adapted on these parameters to get better results.

### P4 The principle of non-surgical treatment of idiopathic scoliosis right-sided breast depending on the volatility of the formation of the intervertebral discs and vertebral bodies

#### Piotr Majcher, Piotr Gawda

##### Uniwersytet Medyczny w Lublinie, Lublin, Poland

**Introduction**

Kinesitherapy procedure in the treatment of children and adolescents with idiopathic scoliosis will vary depending on the size of the angular curvature of the spine and intervertebral krążkó sklinowania size and vertebral bodies, and of whether the child will be treated conservatively, and will prepare for surgery. Rights Delpech-Wolff and Hueter-Volkmann's talk about changes in the growth and remodeling of bone asymmetrically loaded, and the so-called hypothesis. "vicious circle" described by Stroeks talks about the role of the vertebrae in the emerging scoliosis. Is ignored in the literature Polish and international significance of the intervertebral discs in forming the bend.

**Materials and methods**

Research on radiographs of 200 children and adolescents divided into groups at different stages of the creation of the right-hand treatment of thoracic idiopathic scoliosis. Chocking evaluated intervertebral discs and vertebral bodies on the left side compared to the right side of the right-hand idiopathic scoliosis prove that wedged intervertebral discs is essential and may be a prognostic factor in this progresji.Stosunek the amount left to right intervertebral discs and vertebral allows the calculation rate their height.

**Results**

The authors present the principle of non-operative treatment depending on the set values and the degree of curvature angular wedged intervertebral disc and vertebral body in idiopathic scoliosis. Treatment program for children and adolescents with idiopathic scoliosis can be divided according to the value measured by Cobb angle and / or the value of wedged intervertebral disc and vertebral body on:

1. The bending of the spine from 0 to 10 (defect attitude - the attitude of idiopathic scoliosis) no wedged intervertebral disc and vertebral body, recommended only significant physical activity.

2. Curvature of the spine of the angle from 10 to 20-25 and / or wedged vertebral and non-vertebral wedged - recommended intensive individual therapy.

3. Curvature of the spine with a value in excess of 20-25 and / or wedged vertebrae with a value of 0.7 and wedged vertebral body - recommended corrective orthopedic corset, individual therapy.

4. Curvature of the spine above 40-45 and / or wedged vertebrae with a value of 0.54 and wedged vertebral body - recommended surgery, follow-up corrective orthopedic corset, individual therapy fails to improve.

**Conclusions**

1. The treatment of scoliosis is based on their pathomechanics.
2. The study should continue in many centers.

### P5 Unexpected late progression of adolescent idiopathic scoliosis treated with short-term, aggressive, full-time bracing and Schroth physiotherapy with excellent preliminary result: case study

#### Andrea Lebel^1†^, Victoria Ashley Lebel^1,2†^

##### ^1^Ottawa & District Physiotherapy Clinic, Scoliosis Physiotherapy and Posture Centre, McLeod Street, Ottawa, K2P 0Z8, Canada; ^2^Saba University School of Medicine, Saba, Dutch Caribbean, Netherlands.

###### **Correspondence:** Andrea Lebel – Ottawa & District Physiotherapy Clinic, Scoliosis Physiotherapy and Posture Centre, McLeod Street, Ottawa, K2P 0Z8, Canada

^†^These authors contributed equally to this work

**Background**

Adolescent idiopathic scoliosis (AIS) is a complex three-dimensional (3D) spinal deformity of unknown cause diagnosed between the ages of 10 to 18 years. The Scoliosis Research Society (SRS) goal of treatment is to halt curve progression before it reaches surgical magnitude. The risk of scoliosis curve progression decreases with age as the vertical growth potential slows in female AIS patients post-menarche. However, relatively late, unexpected progression of AIS, 1-2 years post-menarche, still has the potential to lead to irreversible structural changes of the spine and torso and to spinal surgery if these patients are only managed with the “wait and see” observation method for scoliosis. The “try and see” method of conservative scoliosis management involving physiotherapeutic scoliosis-specific exercises (PSSE) and 3D bracing should be offered to AIS patients with late, unexpected curve progression, even 1-2 years post-menarche, until the very end of growth. The purpose of this study was to evaluate the effectiveness of conservative management of scoliosis (Schroth physiotherapy and bracing) in a female adolescent patient with late progression of AIS.

**Methods**

This prospective case study follows a 13-year and 10-month old female, 1 year post-menarche, with late progressive AIS, who was treated with short-term, aggressive, full-time bracing and PSSE. The patient was followed by the Schroth physiotherapist from July 2013 to February 2015. The patient’s curve progression and improvement were monitored by measuring vital capacity, chest expansion, and height, in addition to scoliosis curve angle measurements obtained from follow-up radiographs.

**Results**

The case study patient showed significant improvement in vital capacity, chest expansion, and scoliosis curve Cobb angle over the course of this study with short-term, aggressive, full-time bracing and Schroth physiotherapy.

**Conclusions**

In this case study, short-term, aggressive, full-time bracing combined with daily Schroth physiotherapy proved effective in halting curve progression, reducing scoliosis curve Cobb angles, and improving vital capacity, chest expansion, and future quality of life.

**Consent**

Written informed consent was obtained from the patient for publication of this abstract and any accompanying images. A copy of the written consent is available for review by the Editor of this journal.

### P6 Visible posture in relation to the neuroanatomical and neurodynamical features in spinal deformations

#### Piet van Loon^1^, Ruud van Erve^1^, Andre Grotenhuis^2^

##### ^1^Care to Move Orthopedics, Deventer, The Netherlands; ^2^Neurosurgery, UMC Radboud University, Nijmegen, The Netherlands

**Introduction**

All deformities are natural deviations of the optimal posture a human body can achieve. The visible feature of the individual morphology in a standing person is called posture. There is no discussion a normal posture will be formed in gradual ways by forces of neuromuscular origin. So also deformities are caused by these natural forces of growth. The neuro-osseous growth relation and the reciprocal feedback , based on signaling stretch by the nervous cells and moulding capacity of muscular forces on the cartilage ( and young bone) is in depth described by prof. Milan Roth in the period 1965-1985. It reflects the wellknown paradigm: form follows function, which kept its force mainly in paramedical practice and in those Health systems, where prevention of “bad postures”, from the day of birth, kept its place. The relationship between postural deviations and the characteristics of the CNS on MRI is scarcely described.

**Method and material**

In a series of 4 cases with a proven scoliosis or hyperkyphosis a comparison will be shown of all the diagnostic features available to visualize the relationship between external and internal features in spinal deformations. Besides clinical photographs in standing ( and sitting) position and of clinical tests to show the functional signs in postural problems based on neuromuscular thightness, surface topography derived models ( Diers GmbH, Schlangenbad, Germany), standing radiographs and MRI are put alongside to explain the relationships between the main systems involved in deformations during growth: the CNS and the musculoskeletal system.

**Results**

In all cases there is direct relationship between the external form of the spine and the internal features of the central cord/roots complex. Especially the position and the caliber of the central cord and roots in relation to the osseous boundaries of the canal will be shown and explained. The ever present and in fact pathological contact zones between the cord and roots complex at the apices of pathological curves and at the cervicothoracic and the lumbosacral areas present at early ages will be highlighted and related to physiological and pathophysiological signs and symptoms that can occur during adulthood.

**Discussion**

No epidemiologic or cohort study is necessary for understanding pathogenesis , but this visualization of an understandable relationship between external and internal features (most never described in literature) in the involved tissues can invite other institutions with a great number of cases and resources for research on a bigger scale to provide also statistical evidence of the relationships. Concomitant features of a “short cord” like Arnold Chiari malformation and syringomyelie can be explained out of the neuro-osseous growth discongruency.

**Conclusion**

The skeleton and the CNS are strongly related in anatomical and physiological features. An attempt is made to visualize with available diagnostic tools that the anatomic neuro-osseous growth relations that will end-up in spinal deformities if discongruency between the two types of growth occur. The outside always reflects what is going on inside the body.

### P7 Immediate effects of scoliosis-specific corrective exercises on the Cobb angle after 1 week and after 1 year of practice

#### Karina Zapata^1^, Eric Parent^2^, Dan Sucato^1^

##### ^1^Texas Scottish Rite Hospital, Dallas, Texas, USA; ^2^University of Alberta, Edmonton, Canada

**Background**

We are unaware of any studies describing the immediate effects of scoliosis-specific exercises on the Cobb angle measured by radiograph. This study aimed to describe the differences between radiographs obtained with and without corrective exercises after initial training and after one year.

**Design**

Case Study, Level 5

**Methods**

A female with adolescent idiopathic scoliosis was first seen at 13 + 0 years of age with a Risser 0, 2 months post-menarche. She had a 43^o^ left lumbar, 15^o^ right thoracic curve. She had worn a Providence night-time brace for three months. She was seen again after 6, 12 and 24 months and performed exercises from 12 to 24 months. She taught Barcelona Scoliosis Physical Therapy School (BSPTS) exercises for a four-curve type (lumbar dominant with pelvis deviation to the lumbar concave side). She attended 8 visits for 2 hours each over one week of intensive instruction by a BSPTS-certified therapist with 7 months of experience. She was asked to alternate performing 5 of 8 home exercises (semi-hanging, prone-on-knees, prone-on-stool, sidelying, side-sitting, rotational-sitting, sitting, standing) for 30 minutes per day, 5 times a week. No direct care was provided during this interval due to not having access to a trained PT. At 12 and 24 months, x-rays were obtained with and without performing corrective exercises without the PT present.

**Results**

At 6 months, her lumbar and thoracic curves measured 41^o^ and 28^o^, respectively. Her Risser was 2-3 and she had grown 3 cm. At 12 months, her lumbar and thoracic curves measured 47 ^o^ and 30^o^, respectively. Her Risser was 4. The brace was discontinued. Also at 12 months, immediately after her x-ray in the relaxed standing position, she performed her corrective exercises in standing with arms lowered for a second x-ray. The corrections included: pelvis corrections for her curve type, auto-elongation, opening her concavities, depressing her convexities, and shoulder counter-traction. Her lumbar and thoracic curves remained similar and measured 43^o^ and 32^o^, respectively. At 24 months, one year after exercise instruction, her lumbar and thoracic curves measured 26^o^ and 41^o^, respectively. When asked about her improved x-ray, she reported using corrective exercises during the x-ray (without being asked). Another x-ray was obtained in relaxed position during the same visit. Her lumbar and thoracic curves measured 39^o^ and 35^o^, respectively. She was Risser 4 and had grown 3.5 cm the past year. The patient and parents reported home exercise compliance at 1-3 times a week the past year. The immediate effect of corrective exercises after a year of training was a 33 % improvement at the lumbar spine compared to only a 9 % improvement the previous year.

**Conclusion**

After initial training, corrective exercises during a standing x-ray did not significantly improve the Cobb angle for the major lumbar curve compared to the relaxed standing x-ray. However, a year after performing exercises, unsolicited corrective exercises resulted in a significantly improved Cobb angle compared to relaxed standing for the curve primarily targeted by the exercise program. Improved exercise ability and spinal flexibility may have contributed to the improved Cobb angle.

**Consent**

Written informed consent was obtained from the patient for publication of this abstract and any accompanying images. A copy of the written consent is available for review by the Editor of this journal.

### P8 Retrospective analysis of idiopathic scoliosis medical records coming from one out-patient clinic for compatibility with Scoliosis Research Society criteria of brace treatment studies

#### Krzysztof Korbel^1^, Mateusz Kozinoga^2^, Łukasz Stoliński^1^, Tomasz Kotwicki^2^

##### ^1^Rehasport Clinic, Poznań, Poland, ^2^Spine Disorders Unit-Departament of Pediatric Orthopaedics and Traumatology, Poznań University of Medical Sciences, Poznań, Poland

**Background**

According to Scoliosis Research Society (SRS) idiopathic scoliosis (IS) is a spine deformity of more than 10° Cobb angle value with rotation seen on the standing radiograph. In 2005, the SRS experts proposed inclusion criteria for studies on the efficacy of IS corrective brace treatment: a) age ≥10 years, b) Risser 0-2, c) Cobb angle of 25-40°, d) no previous treatment, e) patients before menarche or less than one year after menarche.

**Design and Level of Evidence**

Retrospective study.

**Material and methods**

A retrospective review of the consecutive medical histories of girls with IS treated in the outpatient clinic of the University Hospital in Poznań from 1989 to 2002 was carried out. The outpatient clinic was dedicated to scoliosis problems in general, including congenital and neuromuscular patients. Patients’ age, Cobb angle, Risser sign and menarche status at the beginning of treatment were noted from the charts. The number of patients eligible according to SRS criteria was searched.

**Results**

Total number of 2705 medical charts of consecutive patients (girls only) was checked. 183 out of 2705 girls (6.8 %) undergoing brace treatment were aged ≥10 years and presented Cobb angle in the range 25°-40°. However, considering the maturation status, the number of girls fully following the SRS criteria diminished to 102 girls (3.8 %): 42 presented single thoracic curve and 60 presented double thoracic and lumbar curve. The remaining 81 girls had to be excluded for the following reasons: a) 9 single thoracic; Risser 0-2, 1st menstruation > 1 year, b) 9 single thoracic: Risser sign > 2, 1st menstruation ≤ 1 year, c) 11 single thoracic: Risser sign > 2, 1st menstruation > 1 year, d) 9 double thoracic and lumbar; Risser sign 0-2, 1st menstruation > 1 year, e) 15 double thoracic and lumbar; Risser sign > 2, 1st menstruation ≤ 1 year, f) 28 double thoracic and lumbar; Risser sign > 2, 1st menstruation > 1 year. The mean age at first visit was 12.6 ± 1.7 years, the mean age at start of brace treatment was 12.9 ± 1.7 years.

**Discussion and conclusion**

In the years 1989-2002, the majority of IS brace treated girls were older, more mature and with bigger Cobb angle comparing to current SRS criteria for brace efficacy studies. This study certifies about the changing criteria for conservative treatment of IS. In the light of our findings it seems difficult to directly compare the nowadays results with historical series of cases.

### P9 Adult female with severe progressive scoliosis possibly secondary to benign tumor removal at age 3 treated with scoliosis specific Schroth physiotherapy after refusing surgery: case study

#### Andrea Lebel^1†^, Victoria Ashley Lebel^1,2†^

##### ^1^Ottawa & District Physiotherapy Clinic, Scoliosis Physiotherapy and Posture Centre, McLeod Street, Ottawa, K2P 0Z8, Canada; ^2^Saba University School of Medicine, Saba, Dutch Caribbean, Netherlands

^†^These authors contributed equally to this work

**Background**

Scoliosis is a complex three-dimensional (3D) spinal deformity. Acquired scoliosis in early childhood may progress into adulthood and pose an increased risk of health problems and reduction in quality of life. In Canada, adult patients with scoliosis are not referred for physiotherapeutic scoliosis-specific exercises (PSSE) despite the fact that Schroth physiotherapy, a scoliosis-specific 3D posture training and exercise program, can be effective in reducing pain and improving scoliosis curves, vital capacity, and overall quality of life in scoliosis patients. This case study shows that indeed adult curve progression can be stopped and even reversed with scoliosis specific schroth physiotherapy (SSSPT) in an adult patient with scoliosis.

**Methods**

This is a retrospective case study involving a 23-year-old female scoliosis patient who began an outpatient Schroth physiotherapy exercise program and was initially monitored monthly and then annually for improvement in measurements of angle of trunk rotation (ATR) and chest expansion and improvement in vital capacity measured with incentive spirometry. Photos were taken to document body image periodically throughout Schroth physiotherapy treatment. Additionally, the patient completed SRS-22 quality of life questionnaires every 2 years to evaluate daily function, pain, self-imagine, mental health, and scoliosis management satisfaction.

**Results**

Within one month of beginning SSSPT, the patient reported no more back pain and within 2 months, reported improved breathing. The patient also benefitted from improved chest expansion, scoliosis curve angles (measured in Cobb degrees), vital capacity, ATR, and SRS-22 scores. She became more active and resumed all athletic activity within 8 months of beginning Schroth physiotherapy.

**Conclusions**

Adult scoliosis patients are not routinely referred for PSSE in Canada, even though Schroth physiotherapy, a kind of PSSE, is shown to be effective in this case study. This patient was successfully treated with Schroth physiotherapy. Long-term comprehensive Schroth physiotherapy, to help correct and maintain proper posture in all aspects of daily living, should be part of scoliosis management for adult scoliosis patients in Canada to stop and reverse curve progression and improve overall quality of life.

**Consent**

Written informed consent was obtained from the patient for publication of this abstract and any accompanying images. A copy of the written consent is available for review by the Editor of this journal.

### P10 New aspects of scoliosis therapy planning and monitoring

#### Helmut Diers

##### Research & Development, Schlangenbad, Germany

**Background**

Using SST (Spine & Surface Topography) can significantly reduce the amount of harmful radiation in scoliosis treatment and follow-up during therapy. The examinations are possible during standing and walking conditions.

**Aim**

Purpose of this study is the therapy monitoring based on stato-dynamic measured values to show new aspects of therapy planning, especially scoliosis treatment, and monitoring.

**Design**

Patients according to different age groups and gender were measured with static 3D (habitual standing). The age of the patients was between 18-99. The different age groups have a minimum of 100 participants: 18-45, 46-65, 66-99. The focus was on the following parameters: coronal imbalance, pelvic obliquity, pelvic torsion, vertebral rotation and apical deviation.

**Methods**

SST uses surface topography imaging for 3D back scanning and techniques creating 3D models of the spine without exposing patients to any ionizing radiation. The spine reconstruction model has been used successfully for more than 20 years in evaluation and treatment of patients with spinal deformities such as scoliosis, lordosis and kyphosis. Dynamic spine analysis is using similar algorithms as used in static measurement.

**Results**

Creating new perspectives trough continuous innovation, steady improvement of products and patient specific solutions are important aspects in the organization of therapy and for patient specific solutions. These three conditions are intermeshed like cogs in a machine, and all play a part in maintaining a continuous flow of reliable monitoring mechanisms for personalized care.

**Conclusion**

Normative data offer guidance. The different parameters are closely interacting and when one value changes, there will be influences to others. Monitoring of these sometimes small changes in comparison to the normative data are essential parts when talking about patient individualized supply. Close supervisions and monitoring guarantees quality in therapy organization.

### P11 Outcome of intensive outpatient rehabilitation in an adult patient with M. Scheuermann evaluated by radiologic imaging – a case report

#### Hagit Berdishevsky

##### SchrothNYC, New York, NY, USA

**Background**

No studies examine the efficacy of intensive specific physical therapy (PT) exercises along with brace for the adult with Scheuermann’s kyphosis (SK).

The aim of this study was to examine the effects of intensive PT based on the Barcelona Scoliosis Physical Therapy School (BSPTS) and SpinoMed brace on a 76-year-old female with SK.

**Case Description**

J, 76-year-old female, diagnosed with SK as an adolescent, presented in October 2014 with thoracic hyperkyphosis T1 to T12 Cobb angle of 85° and lumbar hyper lordosis L1 to L5 Cobb angle 70^0^. Lumbar scoliosis T12-L5 with 21^0^ Cobb and vertebral rotation 2. Trunk translation in the sagittal plan was 4.5 cm. Intermittent low back pain 6/10 at worst. Quality-of-life score was 3.8 (SRS 22 questionnaire).

**Method**

The PT regimen included one-hour Schroth exercise sessions three times per week for six months. In addition, a home exercise program (HEP) was recommended. Patient also wore a SpinoMed brace for two hours per day. All tests and measurements were recorded before and after treatment.

**Results**

After a six-month treatment period the kyphosis Cobb angle was reduced to 70° and lordosis improved to 57°. A recent x-ray (October 2015) showed another improvement in the sagittal plane with thoracic kyphosis measuring 64^0^ and lumbar lordosis 55^0^. Lumbar curvature decreased to 12^0^ and vertebral rotation to 1. The quality-of-life score showed improvement with a score of 4.5 on the SRS 22. Pain score diminished to 2. Trunk deviation improved by 2.2 cm.

**Conclusion**

These findings suggest that intensive and specific PT and bracing was successful for the treatment of this adult patient with SK.

**Consent**

Written informed consent was obtained from the patient for treatment, photos, and publication of this case study. A copy of the written consent is available for review by the Editor of this journal.

**References**.

1. Iemolo B: Seven Criteria to Treat Scheuermann’s Disease: Scoliosis. 2007 2(suppl1):S39

2. Wikipedia: Kyphosis. https://en.wikipedia.org/wiki/Kyphosis.

3. Weiss HR, Dieckmann J, Gerner HJ: Outcome of In-patent Rehabilitation in Patients with M. Scheuermann Evaluated by Surface Topography: Studies in Health Technology and Informatics. 2002, 88:246-249

4. Weiss HR, Dieckmann J, Gerner HJ: Effect of Intensive Rehabilitation on Pain in Patients with Scheuermann's disease. Topography: Studies in Health Technology and Informatics. 2002, 88:254-257

5. Sinaki M, Itoi E, Rogers JW, Bergstralh EJ, Wahner HW: Correlation of Back Extensor Strength with Thoracic Kyphosis and Lumbar Lordosis in Estrogen-Deficient Women: *Amer J Phys Med & Rehab.* 1996, 75(5):370-375

6. Pfeifer M, Begerow B, Minne HW: Effects of a new spinal orthosis on posture, trunk strength, and quality of life in women with postmenopausal osteoporosis: A randomized trial: *Am J Phys Med Rehabil.* 2004, 83:177–186

### P12 The effectiveness of a Scoliosis Specific Home Exercise Program and bracing to reduce an idiopathic scoliosis curve with more than 90 % success in less than a year of exercises. Case report

#### Hagit Berdishevsky

##### SchrothNYC, New York, NY, USA

**Background**

There have been no specific studies regarding the effect of an intensive home exercise program (HEP) using the Barcelona Scoliosis Physical Therapy School (BSPTS) method based on the principles of Katharine Schroth.

The aim of this study was to examine the efficacy of an eight-month scoliosis specific HEP combined with the Wood Cheneau Rigo (WCR) brace on an eleven-year-old adolescent girl, A’, with adolescent idiopathic scoliosis (AIS).

**Case Description**

An eleven-year-old girl diagnosed with AIS in 2013. Curvatures were measured: 24.8^0^ Upper thoracic, 41^0^ thoracic, and 21.7^0^ lumbar, Risser 0, angle of trunk rotation (ATR) 10^0^. Trunk imbalance to the thoracic convex side (right), Pelvic translation to the thoracic concave side (left), No pain reported on initial evaluation. Physical therapy using the BSPTS/Schroth method and WCR brace initiated within three and a half months of diagnosis.

**Method**

Patient received ten hours of intensive outpatient physical therapy to learn BSPTS/Schroth-based exercises prior to bracing. This program was followed by an additional 10 hours of instructional sessions. HEP 30-60 min/day, 5 days/week. WCR brace worn 22 hours/day.

**Results**

By the conclusion of the eight-month treatment period, the patient had experienced significant and measurable improvement: Upper thoracic 9.60, Thoracic 00 and lumbar 80. The patient ATR reduced from 10^0^to 2^0^in less than two years.

**Conclusion**

This case study demonstrates that bracing and self-discipline HEP A was able to significantly improve her curve magnitude and clinical appearance in eight months.

**Consent**

Written informed consent was obtained from the patient’s parent for treatment, photos, and publication of this case study. A copy of the written consent is available for review by the Editor of this journal.

**References**

1. Rigo M, Quera-Salvá G, Villagrasa M, Ferrer M, Casas A, Corbella C, Urrutia A, Martínez S, Puigdevall N: Scoliosis intensive out-patient rehabilitation based on Schroth method. *Stud Health Technol Inform*. 2008, 135:208-27

2. Schreiber S, Parent EC, Hedden DM, Hill D, Moreau MJ, Lou E, Watkins EM,Southon SC. The effect of Schroth exercises added to the standard of care on the quality of life and muscle endurance in adolescents with idiopathic scoliosis—an assessor and statistician blinded randomized controlled trial: “SOSORT 2015 Award Winner”. Scoliosis. 2015;10:24.

3. Kuru T, Yeldan İ, Dereli EE, Özdinçler AR, Dikici F, Çolak İ. The efficacy of three-dimensional Schroth exercises in adolescent idiopathic scoliosis: A randomised controlled clinical trial. Clinil Rehabil. 2015.

4. Rigo M ,Villagrasa M, Galo DA: Specific Scoliosis Classification Correlating with Brace Treatment: Description and Reliability. *Scoliosis*. 2010, 5:1

5. Monticone M, Ambrosini E, Cazzaniga D, Rocca B, Ferrante S: Active Self-Correction and Task-Oriented Exercises Reduce Spinal Deformity and Improved Quality of Life in Subjects with Mild Adolscent Idiopathic Scoliosis. Results of Randomised Controlled Trial. *Eur Spine J*. 2014, 23(6):1204-14

